# Biomass-Waste-Derived Silica for Solvent-Lean Cu-SSZ-13 Porous Catalysts in NH_3_-SCR NO_x_ Remediation

**DOI:** 10.3390/molecules31183282

**Published:** 2026-09-16

**Authors:** Marcos Antônio Klunk, Nattan Roberto Caetano, Giulio Lorenzini, Elder Marino Mendoza Orbegoso

**Affiliations:** 1 Geology and Geophysics Research Group—NGA, University of Vale Do Rio Dos Sinos, São Leopoldo 93022750, Brazil; 2 Department of Mechanical Engineering, Federal University of Santa Maria, Santa Maria 97105900, Brazil; 3 Department of Industrial Systems and Technologies Engineering, University of Parma, Parco Area Delle Scienze 181/A, 43124 Parma, Italy; giulio.lorenzini@unipr.it; 4 Department of Mechatronics Engineering, National University of Trujillo, Trujillo 13011, Peru; eldermendoza@unitru.edu.pe

**Keywords:** Cu-SSZ-13, rice husk ash, metakaolin, biomass-waste-derived porous materials, vapor-assisted crystallization, reduced-OSDA synthesis, NH_3_-SCR, NO_x_ remediation

## Abstract

The development of biomass-waste-derived functional porous materials can support resource-conscious strategies for environmental remediation. In this study, Cu-SSZ-13 porous catalysts were prepared from rice husk ash as a biomass-derived silica source and metakaolin as the aluminosilicate precursor using solvent-lean vapor-assisted crystallization under OSDA-free or reduced-OSDA conditions. The S1–S8 catalyst matrix was prepared at nominal Si/Al ratios of 12 and 25, with crystallization conducted in the absence or presence of Cu, followed by a common post-synthetic Cu ion-exchange procedure. XRD showed predominant CHA formation across the matrix, while ^27^Al and ^29^Si MAS NMR confirmed predominantly tetrahedral Al environments and systematic differences in the silicate-framework response. Final Cu contents ranged from 1.50 to 2.61 wt%, with Cu/Al ratios of 0.22–0.27, and samples crystallized in the presence of Cu showed a consistent tendency toward slightly higher final Cu contents than their paired Cu-free-crystallization counterparts. The fresh catalysts exhibited substantial NH_3_-SCR activity, with apparent T_50_ values of 201.3–278.4 °C and maximum NO_x_ conversions of 89.9–97.0%; S4 showed the earliest light-off. Accelerated hydrothermal aging shifted T_50_ by 32.4–65.3 °C while retaining 91.1–94.9% of the fresh maximum NO_x_-conversion response. Estimated N_2_ selectivity remained high throughout most of the principal activity region, whereas relative N_2_O formation remained limited and increased mainly at elevated temperature. Overall, the results demonstrate that a rice-husk-ash/metakaolin precursor platform can be converted into functional CHA-type Cu-SSZ-13 porous catalysts for NO_x_ remediation using OSDA-free or reduced-OSDA, solvent-lean synthesis while retaining substantial catalytic functionality after accelerated hydrothermal exposure.

## 1. Introduction

Zeolites are crystalline microporous aluminosilicates whose framework topology, composition, charge distribution, and local coordination environments influence adsorption, ion exchange, acidity, molecular transport, and catalytic behavior [1]. Their ordered pore systems provide confined environments for charge-balancing cations and exchanged metal species, making zeolites useful platforms for structure–property–function relationships in environmental applications [2].

Among these materials, SSZ-13 is particularly relevant because its chabazite (CHA) topology combines three-dimensional microporosity, eight-membered-ring windows, and confined cages capable of accommodating exchanged metal species [3,4]. Framework composition, pore architecture, structural integrity, and accessibility can influence molecular transport and the stabilization of active environments [5]. In Cu-SSZ-13, framework Al provides negative charge for exchanged Cu species, while final Cu content, Al-associated charge-balancing environments, confinement, and transport collectively influence NH_3_ interaction and catalytic behavior [6].

These characteristics are central to the selective catalytic reduction of nitrogen oxides with ammonia (NH_3_-SCR), an established technology for converting NO_x_ primarily into N_2_ and H_2_O [7]. Despite increasing transport electrification, effective NO_x_-abatement catalysts remain important for heavy-duty and long-distance combustion applications [8,9]. Cu-SSZ-13 is a widely studied CHA-based SCR catalyst because it can combine low-temperature activity, a broad operating range, and substantial hydrothermal resistance under suitable compositions and operating conditions [10,11,12,13]. Its performance is associated with the confinement and accessibility of exchanged Cu species and NH_3_-derived intermediates within the CHA pore system [14,15].

Although Cu-SSZ-13 has been extensively investigated, the extent to which crystallization history influences framework characteristics that subsequently affect Cu incorporation and NH_3_ interaction remains less clearly established. Cu is commonly introduced after crystallization by ion exchange, but the presence of Cu-containing salts during synthesis may also be associated with differences in CHA formation and average framework environments before final Cu exchange [16]. Distinguishing Cu present during crystallization from Cu incorporated into the final catalyst is therefore essential when evaluating synthesis-history effects.

Conventional SSZ-13 preparation frequently relies on high-purity synthetic precursors, substantial quantities of organic structure-directing agents (OSDAs), and liquid-intensive hydrothermal processing [17,18,19,20,21]. These requirements increase material and processing demands [22]. Rice husk ash (RHA) provides a biomass-waste-derived silica source, whereas metakaolin (MK) provides an aluminosilicate precursor [23]. Their combined use offers an alternative route for incorporating waste-derived silica into CHA synthesis while reducing dependence on fully synthetic Si and Al sources [24,25]. A remaining challenge is to combine these precursors with reduced OSDA demand and solvent-lean crystallization while retaining suitable framework characteristics and catalytic functionality.

In the present study, the absence or presence of Cu during crystallization was investigated as a synthesis-history variable rather than as a directly demonstrated catalytic mechanism. Although Cu-containing species may interact with the evolving aluminosilicate precursor, the available characterization does not resolve a unique nucleation pathway or atomistic mechanism [26,27,28]. The main S1–S8 matrix comprised four paired comparisons in which nominal Si/Al ratio, OSDA condition, and crystallization time were maintained within each pair, while Cu was either absent or present during crystallization. All S1–S8 materials were subsequently subjected to the same post-synthetic Cu ion-exchange procedure; accordingly, the terms Cu-free crystallization and Cu-containing crystallization refer exclusively to the crystallization stage.

**Figure 1:**
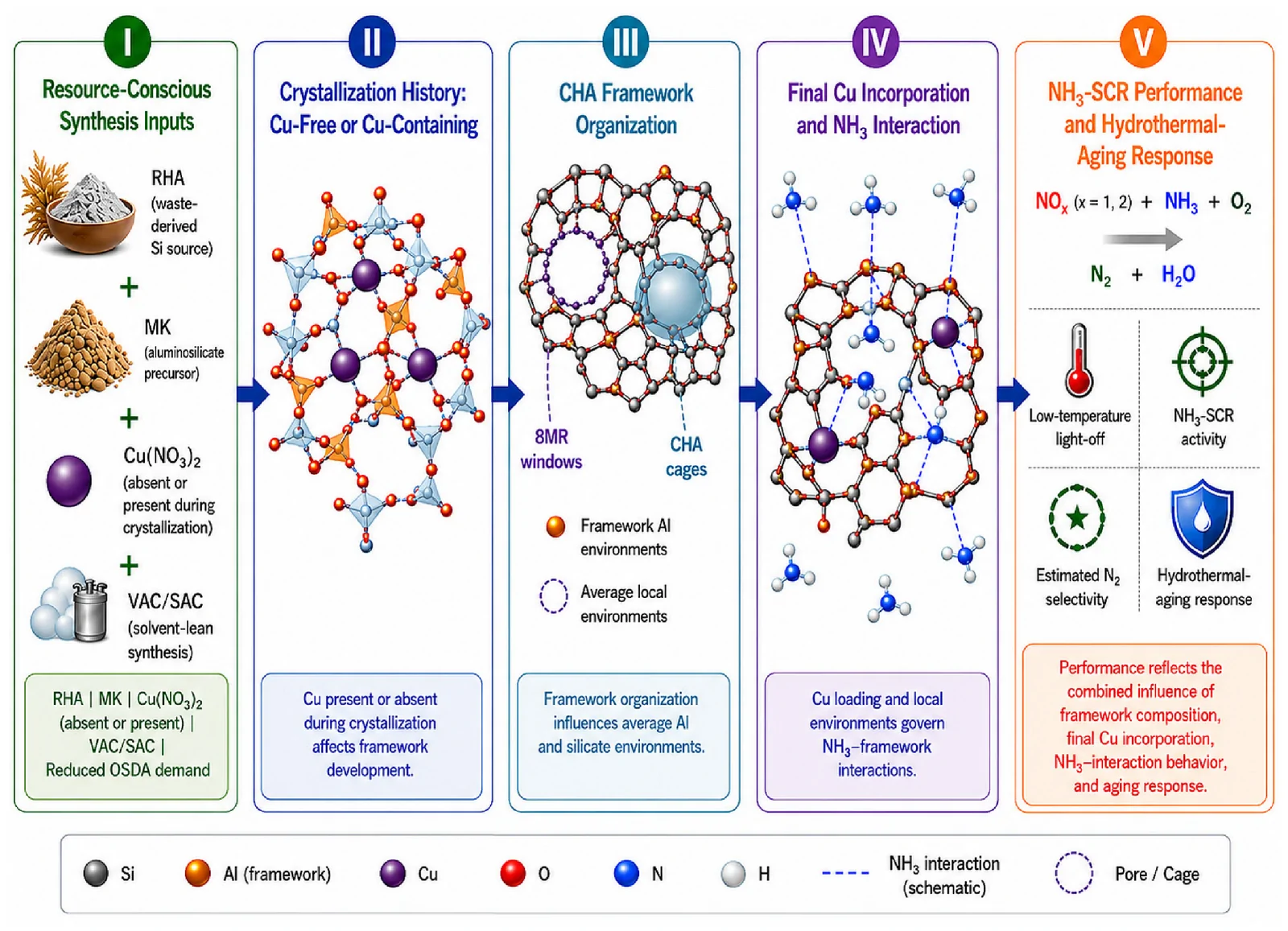
Comparative framework relating synthesis inputs, crystallization history, CHA framework organization, final Cu incorporation, NH_3_-interaction behavior, NH_3_-SCR performance, and hydrothermal-aging response.

The materials were prepared by vapor-assisted crystallization (VAC), also referred to as steam-assisted crystallization (SAC), in which the precursor phase was exposed to water vapor rather than immersed in bulk liquid [29,30]. Here, solvent-lean refers specifically to this crystallization configuration and does not imply a demonstrated overall reduction in environmental impact, which would require dedicated process-level and life-cycle assessment. The overall synthesis–structure–function framework is summarized in Figure 1 [31,32].

This study therefore aimed to investigate the conversion of RHA/MK-derived precursors into Cu-SSZ-13 porous catalysts under OSDA-free or reduced-OSDA, solvent-lean conditions and to evaluate the relationships among CHA framework development, final Cu incorporation, NH_3_-interaction behavior, temperature-programmed NH_3_-SCR performance, nitrogen-product response, and accelerated hydrothermal aging. S10 and S11 were retained as auxiliary preparations corresponding to S4 and S8, respectively, whereas S12 served as a NaNO_3_-containing salt control.

## 2. Results and Discussion

### 2.1. Structural, Compositional, and Functional Characteristics of the RHA/MK-Derived Cu-SSZ-13 Catalysts

The structural characteristics of the RHA/MK-derived materials were evaluated by XRD and ^27^Al and ^29^Si MAS NMR to assess CHA phase formation and the average local environments of Al and Si. The effects associated with nominal Si/Al ratio, OSDA condition, and Cu-free or Cu-containing crystallization were evaluated comparatively across the S1–S8 matrix. Quantitative XRD analysis was restricted to CHA reflection breadth, and no quantitative phase fractions or atomistically resolved framework-site distributions were determined.

S10 and S11 were retained as auxiliary preparations corresponding to S4 and S8, respectively, whereas S12 served as the NaNO_3_-containing salt control corresponding to S2. These auxiliary samples supported interpretation of the synthesis matrix but were not included in the principal S1–S8 structural or catalytic comparisons.

#### 2.1.1. CHA Phase Formation by XRD

**Figure 2:**
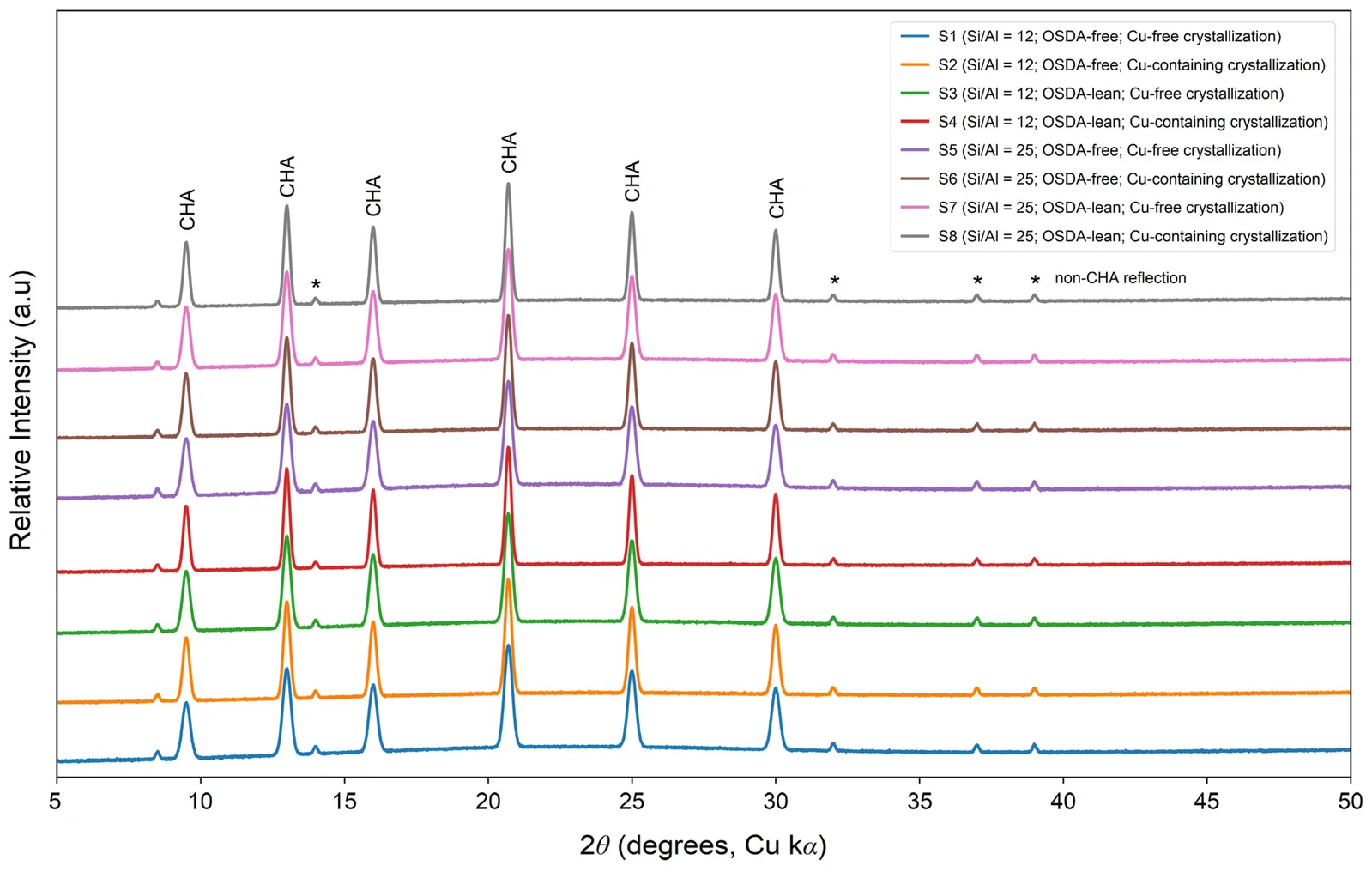
X-ray diffraction patterns of samples S1–S8 prepared from the RHA/MK precursor system under different nominal Si/Al ratios, OSDA conditions, and Cu-free or Cu-containing crystallization histories. The dominant reflections are consistent with the CHA topology, whereas weaker non-CHA reflections are indicated by asterisks (*). These secondary reflections were not assigned to specific crystalline phases because independent phase identification and quantitative phase analysis were not performed.

The XRD patterns of S1–S8 (Figure 2) exhibit the principal reflections characteristic of the CHA topology at approximately 2θ = 9.5°, 13.0°, 16.0°, 20.7°, 25.0°, and 30.0°, indicating that CHA is the predominant crystalline component throughout the RHA/MK-derived catalyst matrix.

Mean FWHM values calculated from these six reflections ranged from 0.240 to 0.331° 2θ. Within each paired comparison, the Cu-containing crystallization member exhibited narrower reflections than the corresponding Cu-free material: 0.270 versus 0.331° for S2/S1, 0.240 versus 0.310° for S4/S3, 0.270 versus 0.320° for S6/S5, and 0.240 versus 0.300° for S8/S7. This consistent paired trend indicates that Cu-containing crystallization was associated with reduced CHA reflection breadth under the investigated conditions. These FWHM differences were used only as comparative diffraction descriptors and were not interpreted as quantitative crystallinity, phase fraction, or crystallite size.

Weaker non-CHA reflections are also present in several patterns and are marked by asterisks in Figure 2. Their presence shows that the materials are not phase-pure CHA; however, the substantially stronger CHA reflections support CHA as the dominant crystalline component. Because the secondary reflections were not independently identified or quantified, no specific phase assignments or phase fractions are proposed.

#### 2.1.2. Framework Aluminum Environments by ^27^Al MAS NMR

**Figure 3:**
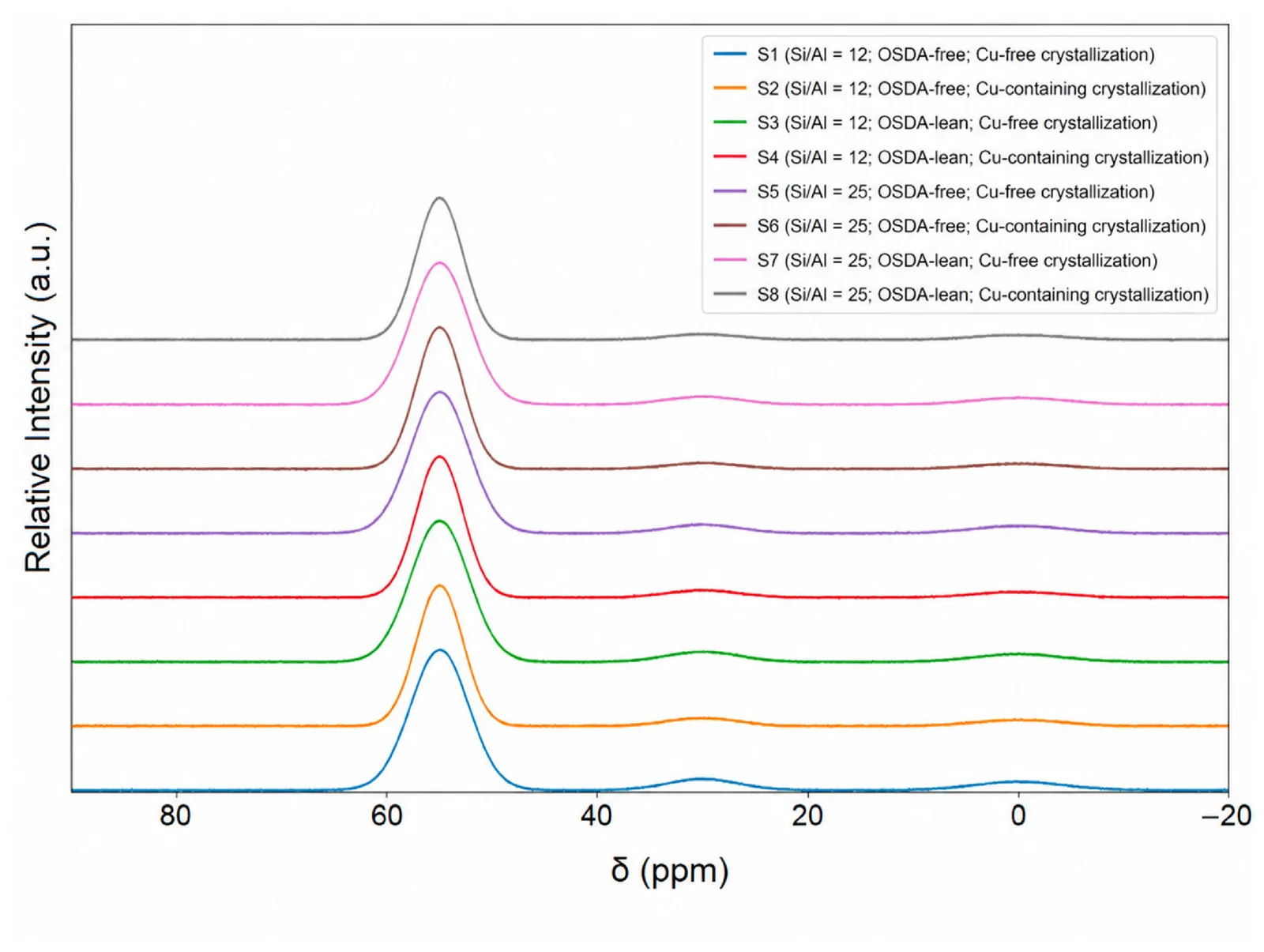
^27^Al MAS NMR spectra of samples S1–S8 after calcination and conversion to the protonic form and before the final post-synthetic Cu ion-exchange step. The dominant resonance near 55 ppm is associated with tetrahedrally coordinated framework Al, Al(IV), whereas weaker contributions in the regions near approximately 25–35 ppm and 0 ppm are consistent with minor non-tetrahedral Al environments. The labels “Cu-free crystallization” and “Cu-containing crystallization” refer exclusively to the absence or presence of Cu during the crystallization stage.

The ^27^Al MAS NMR spectra of S1–S8 are shown in Figure 3. The spectra were recorded after calcination and conversion to the protonic form and before final post-synthetic Cu exchange. All samples are dominated by a resonance near 55 ppm, assigned to tetrahedrally coordinated framework Al, Al(IV), whereas much weaker contributions occur in the intermediate region near 25–35 ppm and around 0 ppm, consistent with minor non-tetrahedral Al environments.

Quantitative integration confirmed the predominance of tetrahedral Al across the S1–S8 matrix (Table 1). The Al(IV) region accounted for 82.3–85.5% of the summed integrated ^27^Al signal, while the intermediate non-tetrahedral and Al(VI) regions each contributed approximately 7–9%. The Al(IV) maxima were narrowly distributed between 54.8 and 55.1 ppm, indicating only limited variation in the position of the dominant tetrahedral-Al resonance across the matrix.

Within each paired comparison, the Cu-containing crystallization member exhibited a slightly higher Al(IV) contribution than its Cu-free counterpart: 84.68 versus 83.06% for S2/S1, 84.06 versus 82.40% for S4/S3, 84.69 versus 82.33% for S6/S5, and 85.47 versus 83.29% for S8/S7. These differences are small, and all samples remained strongly dominated by tetrahedral Al. Accordingly, the regional percentages are interpreted as comparative spectral fractions rather than as absolute populations of uniquely resolved Al species or evidence of specific Al siting or Al-pair distributions.

#### 2.1.3. Silicate Framework Environments by ^29^Si MAS NMR

**Figure 4:**
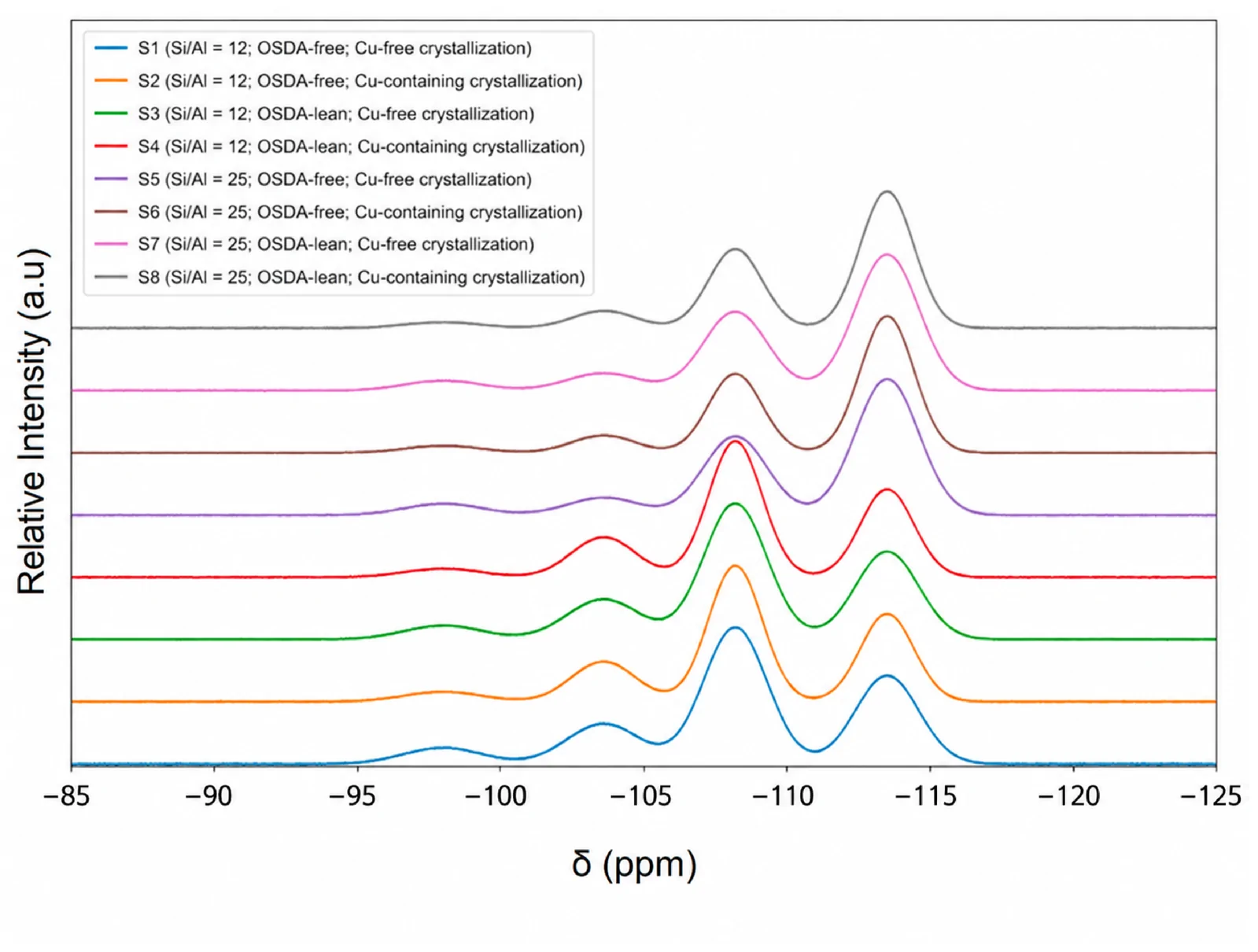
^29^Si MAS NMR spectra of samples S1–S8 after calcination and conversion to the protonic form and before the final post-synthetic Cu ion-exchange step. The broad and overlapping spectral envelopes reflect multiple silicate-framework environments. Quantitative comparison was performed using four constrained Gaussian components; because of spectral overlap and possible defect-related contributions, the fitted components were not assigned uniquely to individual Q^4^(mAl) populations. The labels “Cu-free crystallization” and “Cu-containing crystallization” refer exclusively to the absence or presence of Cu during the crystallization stage.

The ^29^Si MAS NMR spectra of S1–S8 are shown in Figure 4. The spectra were recorded after calcination and conversion to the protonic form and before final post-synthetic Cu exchange. All samples exhibit broad, partially overlapping resonances distributed mainly between approximately −95 and −120 ppm, consistent with multiple Si environments within the aluminosilicate framework. Because of the strong spectral overlap, apparent maxima or shoulders were not assigned directly to individual Q^4^(mAl) populations. In particular, contributions near approximately −102 to −104 ppm may also include defect- or silanol-related Si environments.

To provide a quantitative comparison of the spectral envelopes, the ^29^Si MAS NMR spectra were fitted using four constrained Gaussian components centered approximately near −98, −103 to −104, −108 to −109, and −114 ppm. The relative integrated contributions of these components are summarized in Table 2. In S1–S4, the −108 to −109 ppm component represented 45.8–47.9% of the total fitted signal, whereas the −114 ppm component accounted for 26.4–27.5%. In contrast, S5–S8 showed a larger contribution from the −114 ppm component (53.3–57.1%) and a smaller contribution from the −108 to −109 ppm region (24.4–32.0%).

This systematic shift toward more negative chemical shifts in S5–S8 is consistent with a change in the average silicate-framework environment between the two compositional series. However, because the nominal Si/Al = 12 and Si/Al = 25 series were crystallized for 12 and 18 h, respectively, this difference cannot be attributed exclusively to Si/Al ratio. Within each paired comparison, the Cu-containing and Cu-free crystallization members showed broadly similar fitted distributions. The fitted components are therefore interpreted as comparative spectral contributions and not as uniquely resolved Q^4^(mAl) populations, specific framework sites, or direct evidence of Cu-directed condensation pathways.

#### 2.1.4. Cu-Related Optical Response by UV–Vis DRS

**Figure 5:**
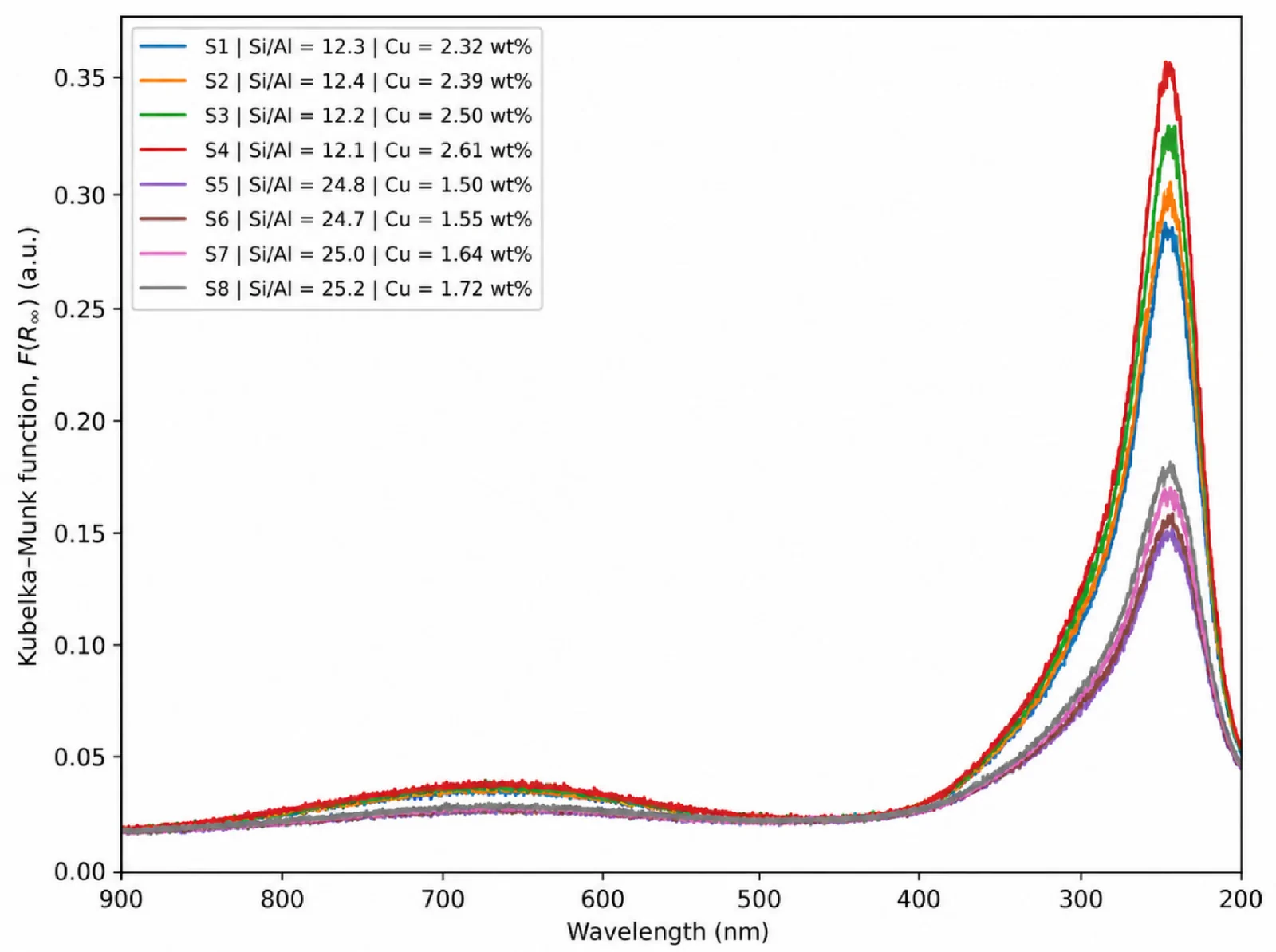
Kubelka–Munk-transformed UV–Vis diffuse reflectance spectra of the S1–S8 Cu-SSZ-13 catalysts after post-synthetic Cu ion exchange and final calcination.

UV–Vis diffuse reflectance spectroscopy was used to compare the Cu-related optical responses of the final post-synthetically Cu-exchanged S1–S8 catalysts (Figure 5). The Kubelka–Munk-transformed spectra exhibit a pronounced UV contribution between approximately 220 and 400 nm and a broader visible-region contribution between approximately 550 and 850 nm [33]. The UV region is consistent with overlapping Cu-related charge-transfer contributions, whereas the broad visible response is consistent with d–d transitions of Cu^2+^-containing environments. Because these bands are broad and partially overlapping, they were not assigned to unique Cu populations, coordination geometries, or oxidation-state distributions.

Quantitative analysis showed principal UV maxima narrowly distributed between 246.2 and 249.8 nm. Baseline-corrected integration yielded UV-region areas of 9.07–11.17 a.u.·nm for S1–S4 and 4.71–5.79 a.u.·nm for S5–S8, whereas the corresponding visible-region areas ranged from 1.92–2.03 and 1.72–1.78 a.u.·nm, respectively. The stronger UV response of S1–S4 accompanied their higher final Cu contents (2.32–2.61 wt%) relative to S5–S8 (1.50–1.72 wt%). Because the two compositional series were crystallized for different durations, this contrast was not attributed exclusively to nominal Si/Al ratio.

Within each paired comparison, the Cu-containing crystallization member showed a modestly larger integrated UV response than its Cu-free counterpart: 9.60 versus 9.07 a.u.·nm for S2/S1, 11.17 versus 10.23 a.u.·nm for S4/S3, 4.89 versus 4.71 a.u.·nm for S6/S5, and 5.79 versus 5.31 a.u.·nm for S8/S7. These changes accompanied corresponding modest increases in final Cu loading and were therefore interpreted as associations among synthesis history, final composition, and Cu-related optical response rather than as isolated effects of Cu presence during crystallization.

S4 exhibited the largest integrated UV response and the highest final Cu content and Cu/Al ratio within S1–S8 (2.61 wt% Cu; Cu/Al = 0.27). It also showed the earliest fresh NH_3_-SCR light-off (T_50_ = 201.3 °C), providing the clearest correspondence in the present dataset between final Cu incorporation, Cu-related optical response, and low-temperature catalytic activation. This relationship remains comparative and does not identify a unique Cu-site configuration.

#### 2.1.5. NH_3_-Interaction Behavior by NH_3_-TPD

**Figure 6:**
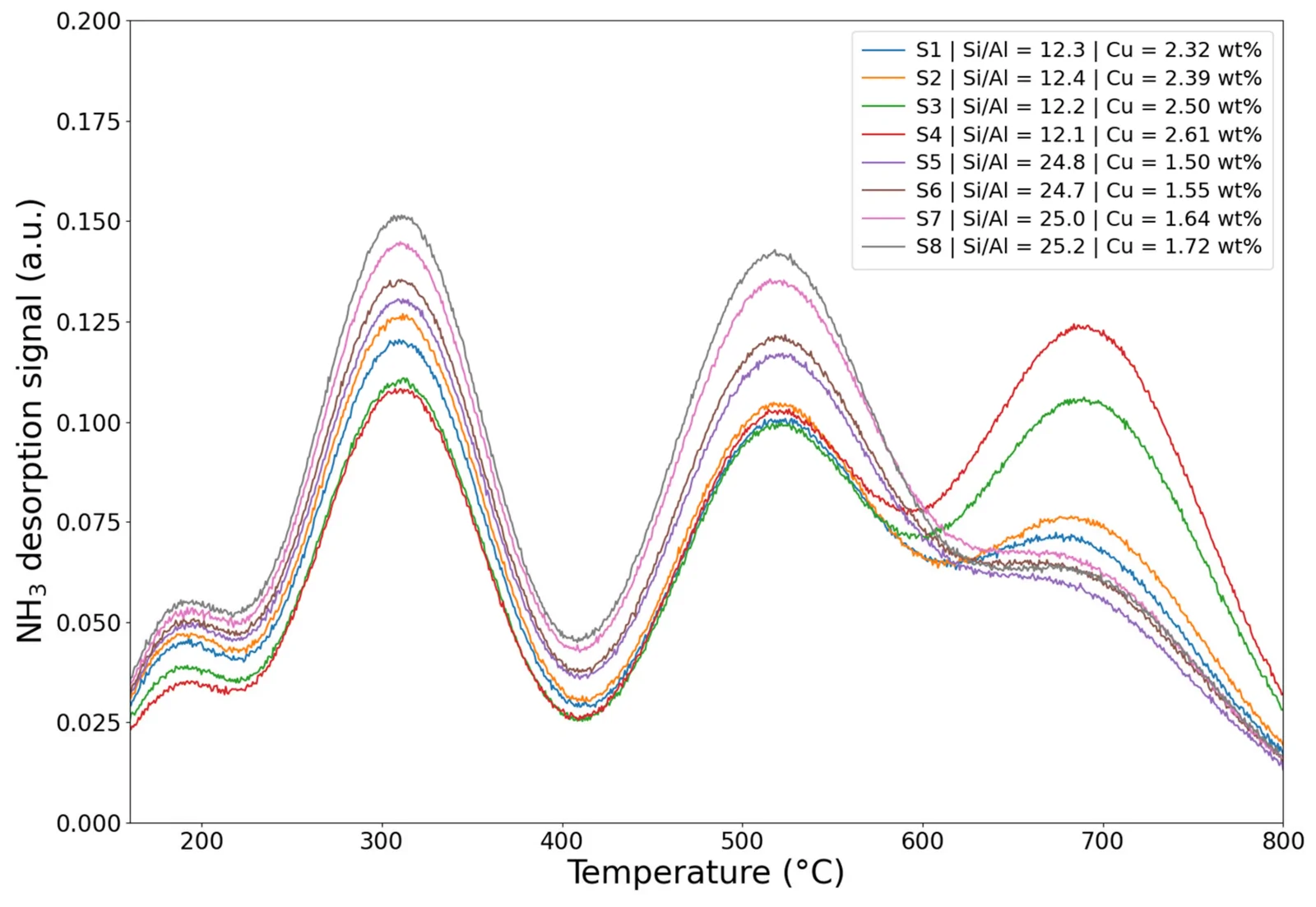
NH_3_-TPD profiles of the final Cu-exchanged S1–S8 catalysts recorded during programmed heating from 160 to 800 °C. The broad and partially overlapping desorption contributions are interpreted comparatively as apparent NH_3_-interaction regions. The profiles are used to examine associations with final Si/Al ratio, Cu content, OSDA condition, and crystallization history and should not be assigned uniquely to individual Brønsted, Lewis, or Cu-associated sites.

The NH_3_-TPD profiles of the final Cu-exchanged S1–S8 catalysts are shown in Figure 6. All samples exhibit broad and partially overlapping desorption contributions, with principal responses centered near approximately 300–320 °C and 500–530 °C. These regions are consistent with NH_3_ retained in environments of different apparent interaction strengths within the CHA microporous system, but they were not assigned uniquely to individual Brønsted, Lewis, or Cu-associated sites because desorption temperature may also be influenced by confinement, transport, readsorption, framework composition, and final Cu content [34].

Quantitative integration of the baseline-corrected profiles is summarized in Table 3. Total relative desorption areas ranged from 27.05 to 35.23 a.u.·°C across S1–S8. Within each paired comparison, the Cu-containing crystallization member exhibited a modestly higher integrated response than its Cu-free counterpart, corresponding to increases of 2.4% for S1/S2, 7.1% for S3/S4, 3.6% for S5/S6, and 3.0% for S7/S8. The principal low-temperature T_max_ values remained narrowly distributed between approximately 309 and 312 °C.

The regional signal distribution showed a clearer compositional contrast. S1 and S2 exhibited nearly equal low- and intermediate-temperature contributions (~38% each), whereas the high-temperature contribution increased to 34.7 and 38.3% for S3 and S4, respectively. In contrast, S5–S8 displayed larger intermediate-temperature contributions of 42.5–44.6% and smaller high-temperature contributions of 16.0–18.6%. Because the Si/Al = 12 and Si/Al = 25 series were crystallized for different durations, these differences were interpreted as combined effects of composition and synthesis history rather than as isolated Si/Al effects.

Overall, the NH_3_-TPD results demonstrate synthesis-dependent differences in both the magnitude and temperature distribution of retained NH_3_ across the S1–S8 matrix. However, the integrated areas are relative signal descriptors rather than absolute acid-site concentrations, and the observed differences were not used to infer unique adsorption-site populations or isolated effects of a single synthesis variable.

#### 2.1.6. Catalytic Performance in NH_3_-SCR Fixed-Bed Tests

**Figure 7:**
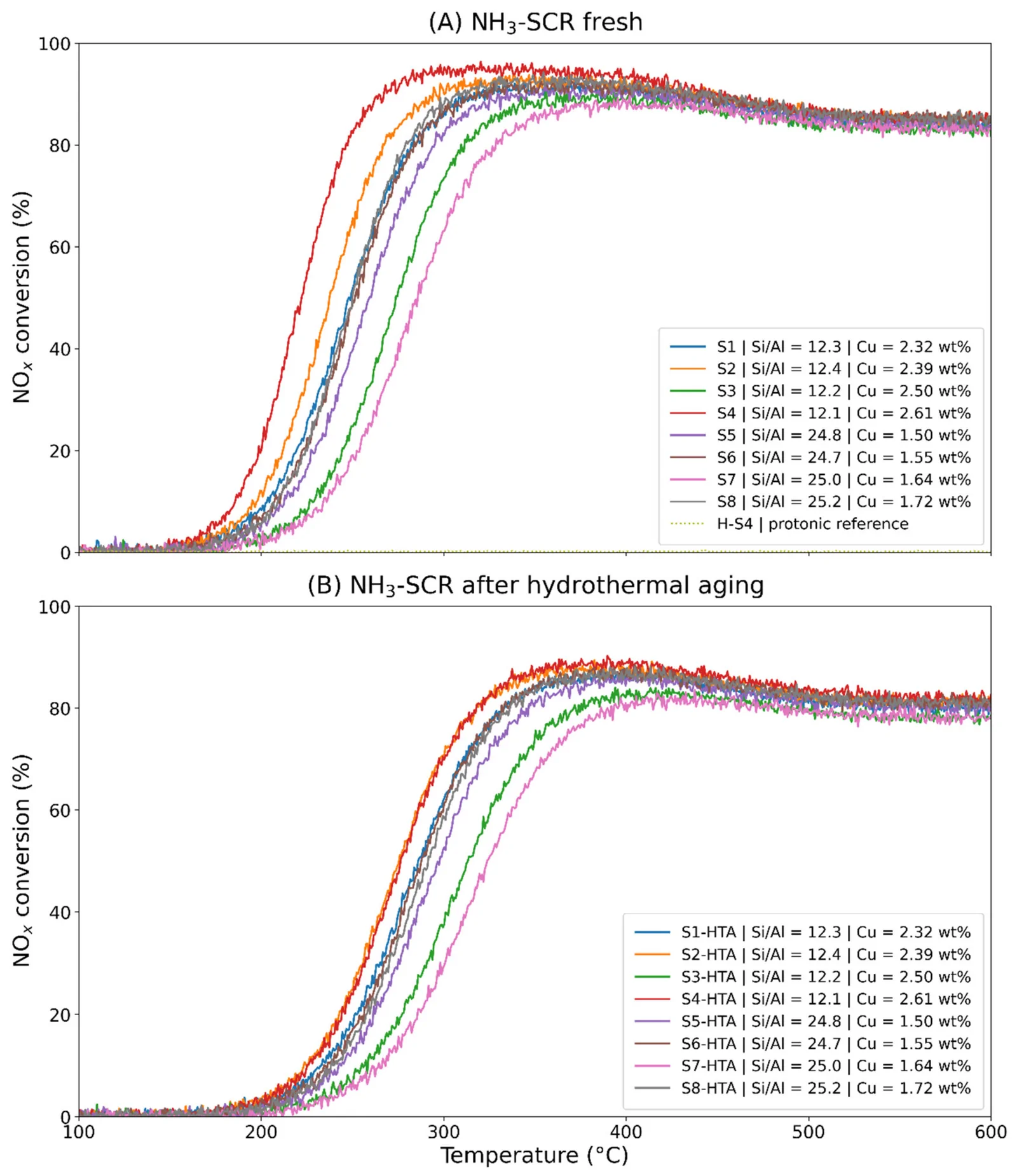
Temperature-programmed NH_3_-SCR NO_x_-conversion profiles of the final Cu-exchanged catalyst matrix under dry-feed fixed-bed conditions: (**A**) fresh catalysts and (**B**) catalysts after accelerated hydrothermal aging. The profiles were recorded during continuous heating from 150 to 600 °C and are interpreted as comparative light-off responses rather than as steady-state catalytic performance.

The temperature-programmed NH_3_-SCR NO_x_-conversion profiles of the final Cu-exchanged S1–S8 catalysts are shown in Figure 7 for the fresh (Figure 7A) and hydrothermally aged (Figure 7B) states. Because the measurements were obtained during continuous heating at 5 °C min^−1^, the profiles are interpreted as comparative light-off responses rather than as steady-state kinetic performance.

All fresh catalysts exhibited substantial NO_x_ conversion, with apparent T_50_ values ranging from 201.3 to 278.4 °C and maximum conversions of 89.9–97.0% (Table 4). S4 showed the earliest light-off (T_50_ = 201.3 °C) and the highest maximum NO_x_ conversion (97.0%), whereas S7 exhibited the latest fresh light-off (T_50_ = 278.4 °C). The remaining catalysts showed intermediate behavior. H-S4, evaluated as the protonic reference before final Cu exchange, exhibited negligible NO_x_ conversion, supporting the central role of the final Cu-containing catalyst state in the observed NH_3_-SCR response.

Accelerated hydrothermal aging shifted the light-off response toward higher temperature for all S1–S8 catalysts. Aged T_50_ values ranged from 263.5 to 312.8 °C, corresponding to ΔT_50_ values of 32.4–65.3 °C. S4 showed the largest shift, from 201.3 to 266.6 °C (ΔT_50_ = 65.3 °C), whereas the other catalysts exhibited shifts of 32.4–42.2 °C. Despite these changes, aged maximum NO_x_ conversion remained between 82.1 and 89.2%, corresponding to retention of 91.1–94.9% of the fresh maximum response.

Thus, under the applied accelerated aging conditions, the principal catalytic effect was a systematic delay in light-off accompanied by a smaller decrease in maximum NO_x_ conversion. No single aging trend could be attributed exclusively to nominal Si/Al ratio, OSDA condition, or crystallization history across the paired matrix. The results therefore demonstrate retained catalytic functionality after hydrothermal exposure without implying unchanged framework structure, Cu-site distribution, or long-term durability under realistic exhaust conditions.

#### 2.1.7. Nitrogen-Product Response and N_2_O Formation

**Figure 8:**
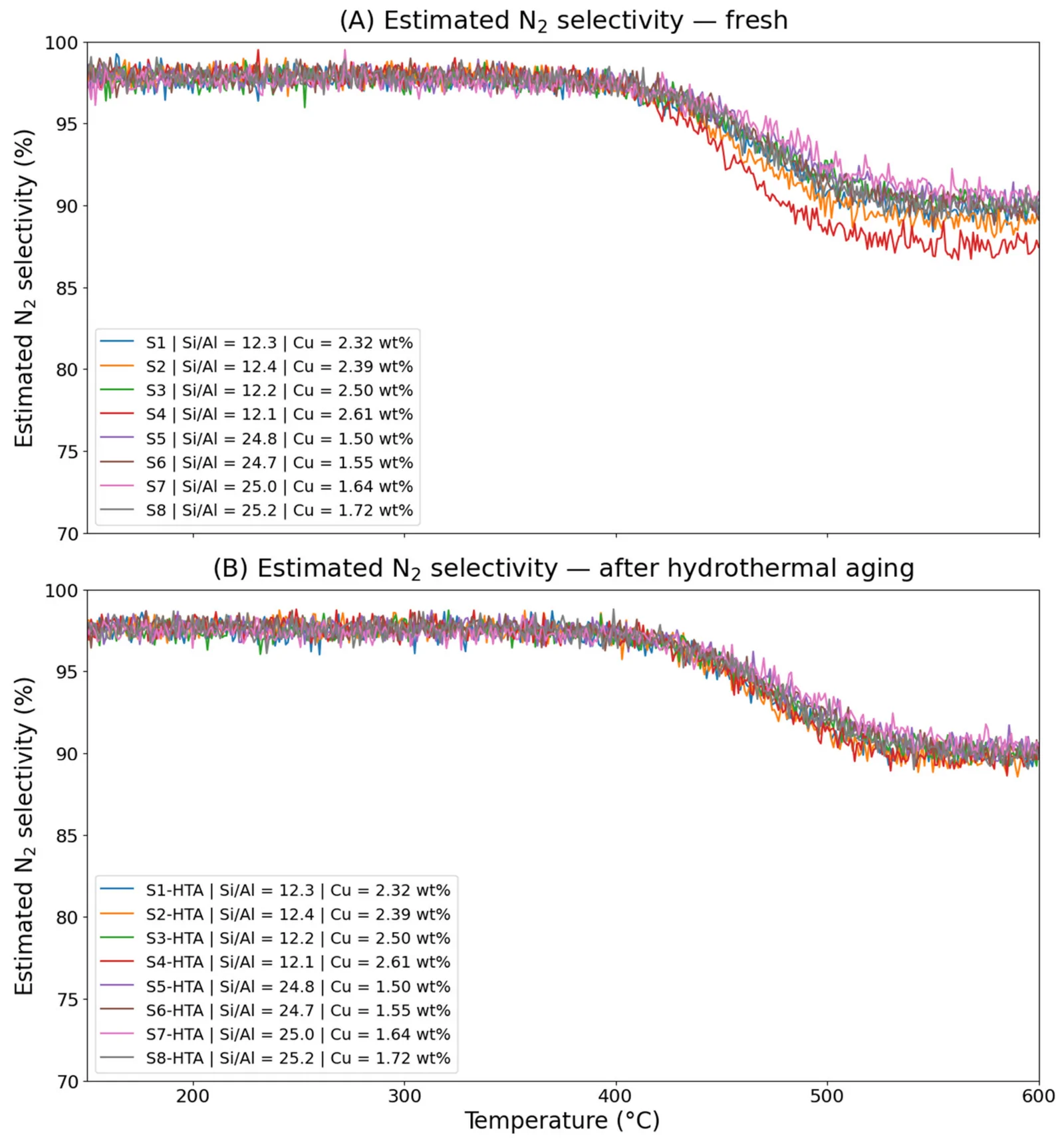
Estimated N_2_ selectivity profiles of the S1–S8 Cu-SSZ-13 catalyst matrix during temperature-programmed NH_3_-SCR testing: (**A**) fresh catalysts and (**B**) catalysts after accelerated hydrothermal aging. N_2_ selectivity was estimated from the atomic nitrogen balance because N_2_ was not measured directly.

**Figure 9:**
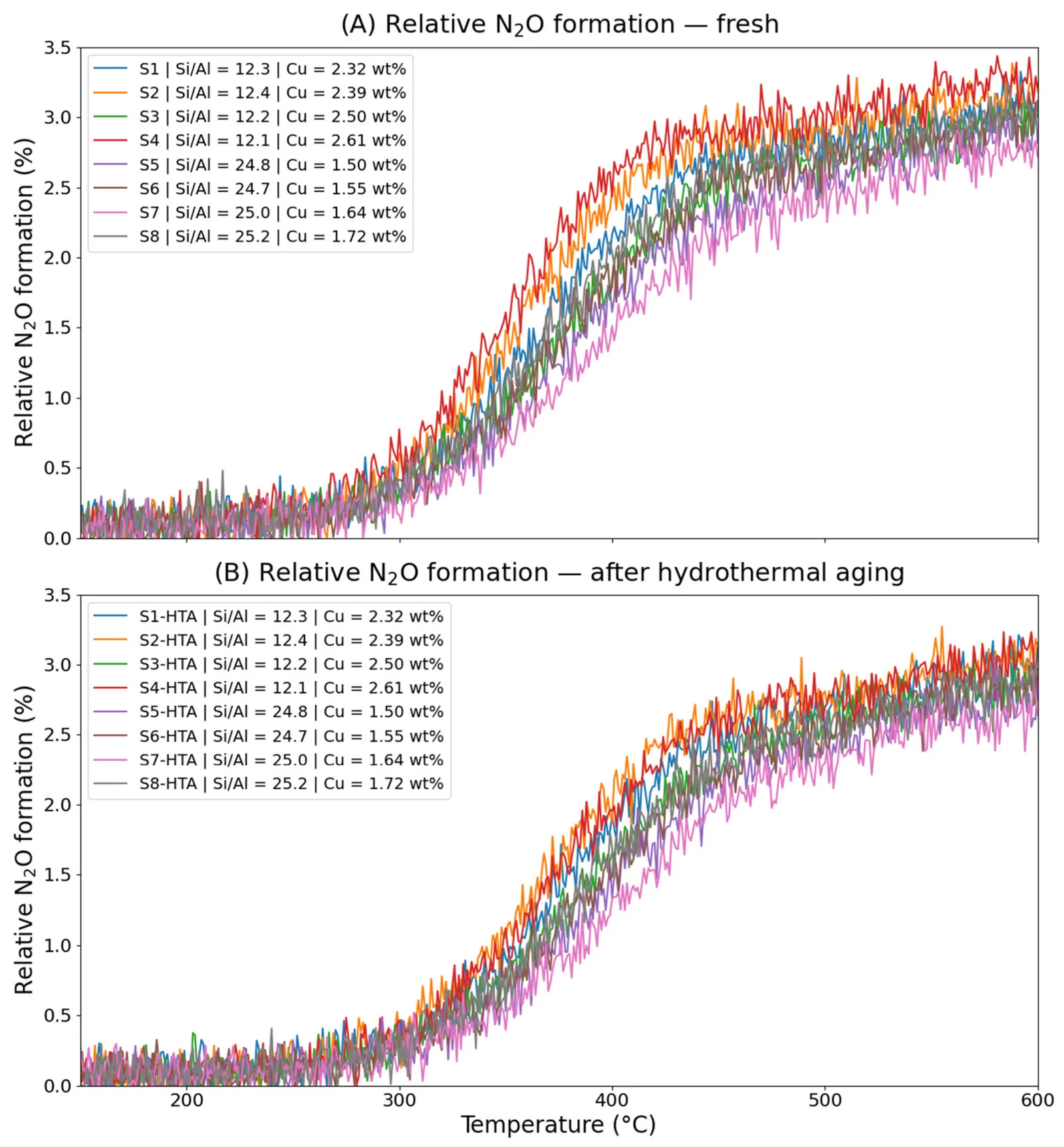
Relative N_2_O formation profiles of the S1–S8 Cu-SSZ-13 catalyst matrix during temperature-programmed NH_3_-SCR testing: (**A**) fresh catalysts and (**B**) catalysts after accelerated hydrothermal aging. Relative N_2_O formation was normalized to the inlet NO_x_ concentration and was treated as a processed descriptor rather than as N_2_O selectivity.

To complement the NO_x_-conversion analysis, the nitrogen-product response of the S1–S8 catalyst matrix was examined using estimated N_2_ selectivity and relative N_2_O formation as processed descriptors. Figure 8 compares the estimated N_2_-selectivity profiles of the fresh and hydrothermally aged catalysts, whereas Figure 9 presents the corresponding relative N_2_O-formation profiles. These descriptors were evaluated separately from the light-off and activity-retention parameters discussed in Section 2.1.6.

For the fresh catalysts, estimated N_2_ selectivity remained generally above approximately 97% throughout most of the principal low- and intermediate-temperature activity region and decreased progressively at higher temperature, particularly above approximately 420–470 °C. At 600 °C, the final estimated values remained broadly within the high-80 to low-90% range. Hydrothermally aged catalysts showed the same general temperature dependence, with comparatively modest changes in estimated N_2_ selectivity relative to the more pronounced light-off shifts observed after aging.

Relative N_2_O formation remained close to zero at low temperature and increased progressively toward the high-temperature region. For the fresh catalysts, relative N_2_O formation reached approximately 2.5–3.0% at 600 °C, with only modest differences among samples. The aged catalysts exhibited broadly similar profiles, and no systematic increase in high-temperature relative N_2_O formation was observed across the complete S1–S8 matrix.

Because N_2_ was not measured directly, the N_2_-selectivity values are estimates obtained from the atomic nitrogen balance. Relative N_2_O formation was normalized to inlet NO_x_ and is not equivalent to N_2_O selectivity based on reacted nitrogen. Accordingly, estimated N_2_ selectivity and relative N_2_O formation have different denominators, were not treated as complementary fractions of a closed nitrogen balance, and were not constrained to sum to 100%.

Overall, the nitrogen-product descriptors indicate that the S1–S8 catalysts maintained high estimated N_2_ selectivity and limited relative N_2_O formation throughout most of the principal NH_3_-SCR activity region before and after accelerated hydrothermal aging. The high-temperature decrease in estimated N_2_ selectivity together with increasing relative N_2_O formation indicates growing competition among nitrogen-conversion pathways, although the present measurements do not resolve the individual reactions responsible.

### 2.2. Integrated Structure–Property–Function Relationships

The combined dataset supports a comparative structure–property–function interpretation for the RHA/MK-derived Cu-SSZ-13 catalyst matrix. Predominant CHA formation was obtained across S1–S8 under OSDA-free and OSDA-lean conditions, while ^27^Al and ^29^Si MAS NMR showed predominantly tetrahedral Al environments and systematic differences in the average silicate-framework response. These structural characteristics were accompanied by successful post-synthetic Cu incorporation, measurable NH_3_ retention, substantial NH_3_-SCR activity, and retained catalytic functionality after accelerated hydrothermal aging.

Final bulk Si/Al ratios remained within 12.1–12.4 for S1–S4 and 24.7–25.2 for S5–S8, while final Cu contents ranged from 1.50 to 2.61 wt% and Cu/Al ratios from 0.22 to 0.27 (Table 5). Within each paired synthesis condition, the Cu-containing crystallization member showed a slightly higher final Cu content than its Cu-free counterpart. However, these differences were small and were treated as a consistent descriptive association rather than as evidence that crystallization-stage Cu directly controlled subsequent Cu uptake.

The functional results further indicate that no single compositional or spectroscopic descriptor independently determined catalytic behavior. Integrated UV–Vis responses generally increased with final Cu content within the paired comparisons, whereas NH_3_-TPD revealed differences in both the magnitude and temperature distribution of retained NH_3_. Nevertheless, fresh NH_3_-SCR light-off did not scale monotonically with either total Cu content or NH_3_-retention intensity. The catalytic response is therefore more consistently interpreted as the combined outcome of framework composition, final Cu incorporation, NH_3_ interaction, synthesis history, and transport within the CHA microporous system.

Accelerated hydrothermal aging shifted T_50_ by 32.4–65.3 °C while retaining 91.1–94.9% of the fresh maximum NO_x_-conversion response. Changes in estimated N_2_ selectivity and relative N_2_O formation were comparatively smaller, indicating that the principal functional effect of aging was delayed catalytic activation rather than a major alteration of the overall nitrogen-product response. At elevated temperature, the parallel decrease in estimated N_2_ selectivity and increase in relative N_2_O formation indicate increasing competition among nitrogen-conversion pathways, although the individual reactions responsible were not independently resolved.

Comparison with representative Cu-SSZ-13 systems reported in the literature (Table 6) shows that several optimized catalysts exhibit earlier light-off, broader high-conversion windows, or stronger hydrothermal performance than the present RHA/MK-derived materials [12,18,19,29,35,36,37].

Accordingly, the present study does not claim catalytic superiority. Its contribution lies instead in demonstrating that biomass-waste-derived silica and metakaolin can be integrated with OSDA-free or reduced-OSDA formulations and solvent-lean vapor-assisted crystallization while retaining substantial NH_3_-SCR functionality.

**Figure 10:**
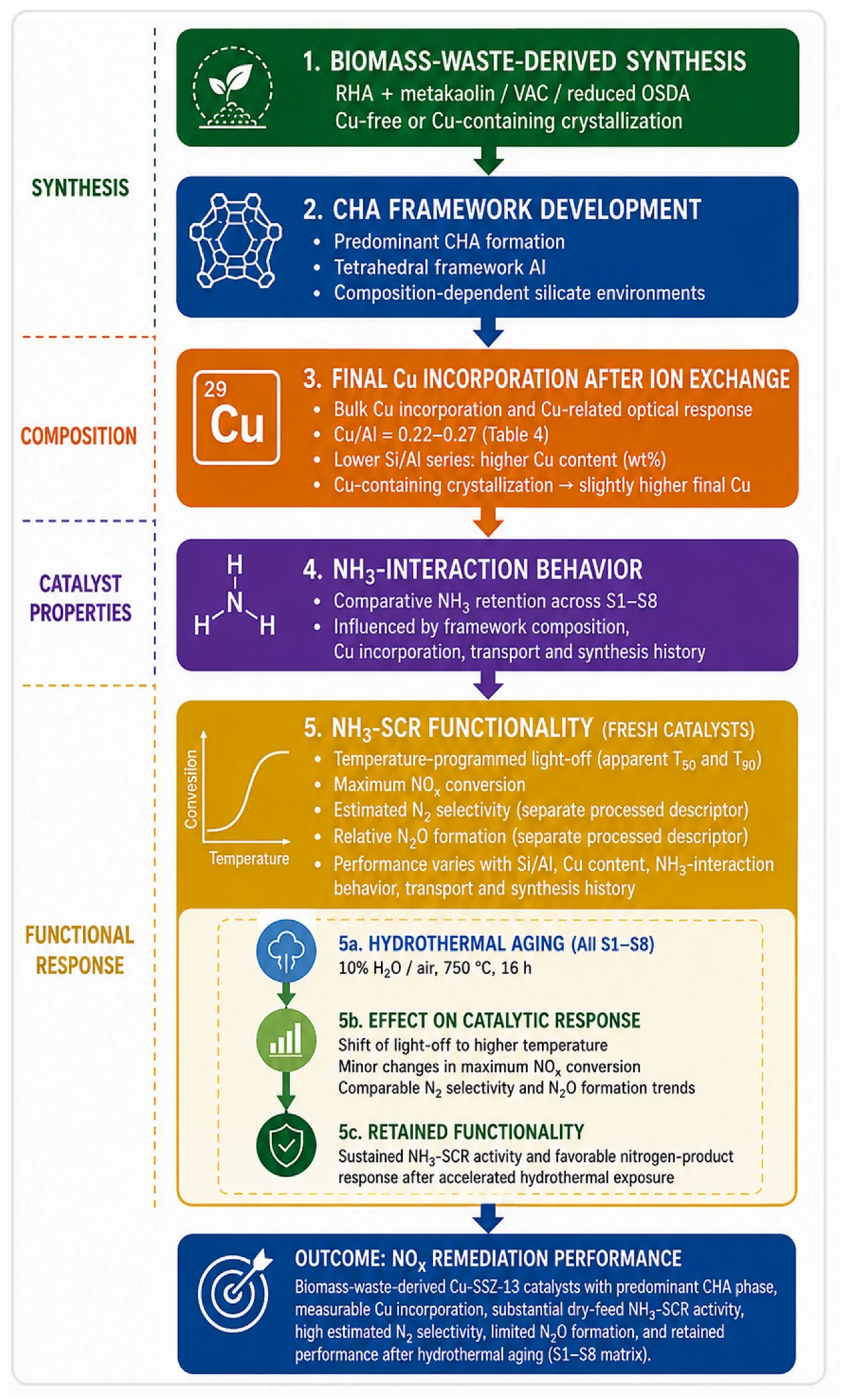
Integrated synthesis–framework–property–function interpretation for the RHA/MK-derived Cu-SSZ-13 catalyst matrix, linking synthesis conditions, CHA framework development, final Cu incorporation, NH_3_ interaction, catalytic performance, nitrogen-product response, and hydrothermal behavior. The scheme represents empirical comparative relationships rather than a unique causal or atomistically resolved mechanism.

The overall relationships are summarized in Figure 10. The scheme represents an empirical synthesis–framework–property–function interpretation linking precursor selection and synthesis conditions to CHA formation, final Cu incorporation, NH_3_ interaction, catalytic response, nitrogen-product behavior, and hydrothermal stability. It should not be interpreted as evidence of a unique causal pathway or atomistically resolved crystallization mechanism.

## 3. Materials and Methods

### 3.1. Starting Materials

Rice husk ash (RHA) was used as the biomass-waste-derived Si source. Before synthesis, it was dried at 105 °C for 24 h and sieved below 75 μm [38,39]. Its composition was 99.08 wt% SiO_2_, 0.09 wt% Al_2_O_3_, 0.04 wt% Fe_2_O_3_, 0.68 wt% K_2_O, and 0.04 wt% SO_3_. Metakaolin (MK) was obtained by calcining kaolin at 750 °C for 4 h at 5 °C min^−1^ [40] and contained 51.45 wt% SiO_2_, 39.97 wt% Al_2_O_3_, 2.09 wt% Fe_2_O_3_, 0.07 wt% Na_2_O, 0.35 wt% K_2_O, 2.66 wt% CaO, 0.67 wt% MgO, and 2.78 wt% TiO_2_.

The RHA and MK masses were calculated from their measured SiO_2_ and Al_2_O_3_ contents to obtain nominal atomic Si/Al ratios of 12 and 25. Sodium hydroxide (NaOH, ≥98%) was used as the alkaline activator, Cu(NO_3_)_2_·3H_2_O for Cu-containing crystallization and post-synthetic Cu exchange, TMAdaOH as the organic structure-directing agent in OSDA-lean formulations, NaNO_3_ for the auxiliary salt-control preparation, and NH_4_Cl for ammonium ion exchange. All reagents were obtained from a Merck/Sigma-Aldrich distributor in Porto Alegre, Brazil. Unless otherwise specified, reagent purities were those reported by the suppliers.

### 3.2. Experimental Design and Synthesis Matrix

The main S1–S8 matrix comprised four paired synthesis conditions designed to compare the absence or presence of Cu during crystallization under two OSDA conditions and two nominal atomic Si/Al ratios. S1/S2 and S5/S6 were OSDA-free, whereas S3/S4 and S7/S8 were OSDA-lean (TMAdaOH/Si = 0.035); within each pair, Cu was either absent or introduced as Cu(NO_3_)_2_·3H_2_O after alkaline activation and before dry-gel formation.

**Figure 11:**
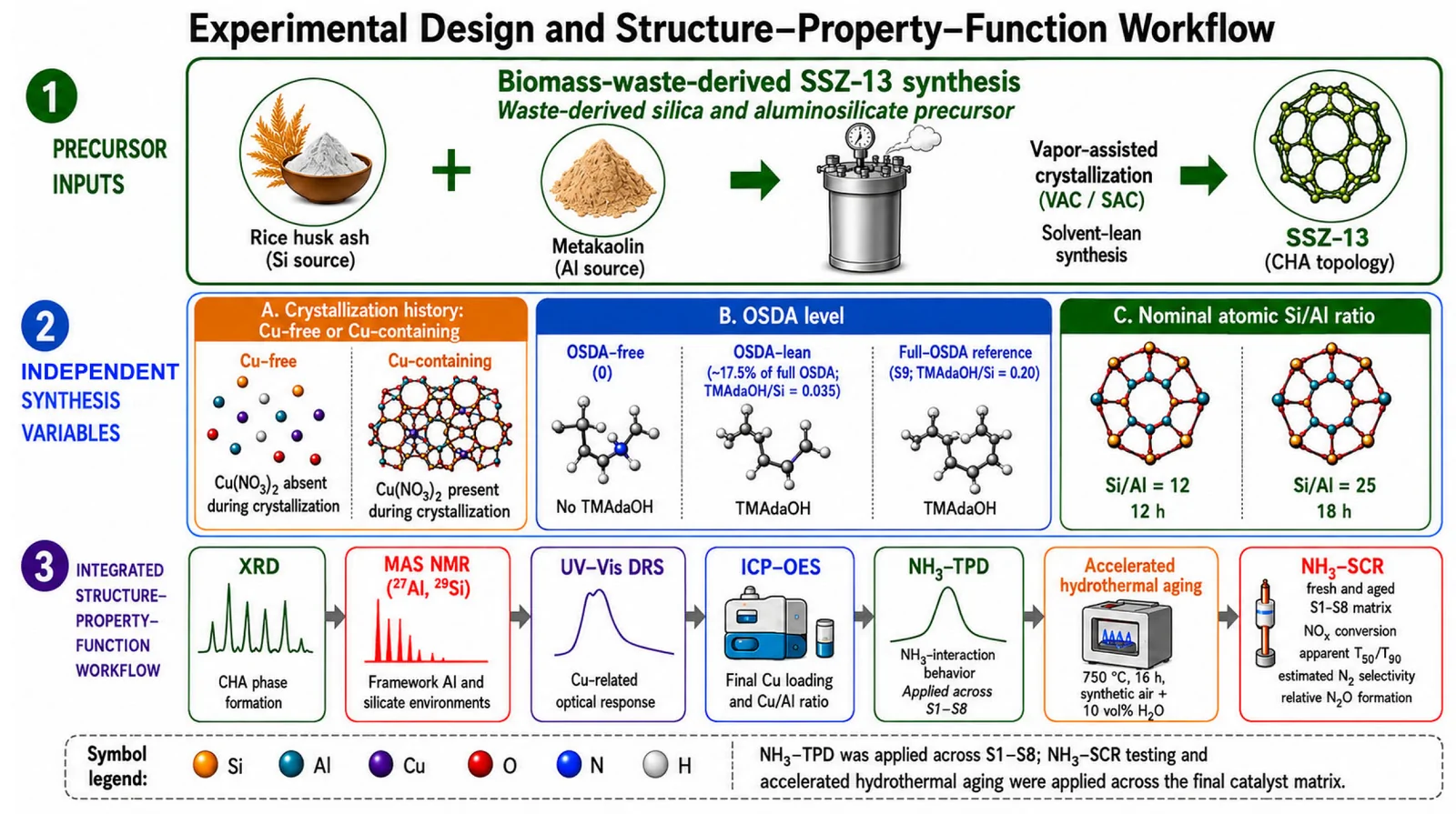
Experimental design and structure–property–function workflow linking the RHA/MK precursor system, crystallization history, OSDA condition, and nominal Si/Al ratio to structural and compositional characterization, NH_3_-interaction analysis, fresh and aged NH_3_-SCR performance, and accelerated hydrothermal-aging response.

The nominal Si/Al ratio was 12 for S1–S4 and 25 for S5–S8. These series were crystallized for 12 and 18 h, respectively; therefore, comparisons between the two compositional groups reflect the combined influence of nominal Si/Al ratio and crystallization duration and were not interpreted as isolated Si/Al effects. The overall experimental workflow is summarized in Figure 11, while the complete sample-role and analytical matrix and the detailed synthesis formulations are provided in Tables S1 and S2, respectively.

### 3.3. Gel Preparation and Alkaline Activation

The RHA/MK precursor formulations were prepared at nominal atomic Si/Al ratios of 12 and 25 using the precursor quantities listed in Table S2. The solids were dispersed in 70 mL of 1.0 mol L^−1^ NaOH solution and magnetically stirred for 2 h at room temperature to promote homogenization and alkaline activation before dry-gel formation. No TMAdaOH was added to the OSDA-free formulations, whereas the OSDA-lean formulations contained TMAdaOH/Si = 0.035, calculated from the total nominal Si content. All precursor masses were weighed within a relative tolerance of ±0.1%.

### 3.4. Cu-Containing Crystallization Conditions

For Cu-containing crystallization, 0.07 mol L^−1^ Cu(NO_3_)_2_ solution was added after the 2 h alkaline activation step and before dry-gel formation. The corresponding initial Cu/Al ratios were approximately 0.047 for the nominal Si/Al = 12 formulations and 0.041 for the nominal Si/Al = 25 formulations; the sample-specific solution volumes are reported in Table S2. These values refer only to Cu introduced during crystallization and are distinct from the final Cu/Al ratios obtained after post-synthetic ion exchange.

The presence or absence of Cu during crystallization was treated as a synthesis-history variable within the paired RHA/MK-based VAC/SAC formulations [41,42,43]. Sample S12 was prepared as an auxiliary NaNO_3_-containing salt control corresponding to S2, using 1.79 mmol NaNO_3_ in place of Cu(NO_3_)_2_ to approximate the added nitrate/salt contribution without introducing Cu.

### 3.5. Dry-Gel Formation and Vapor-Assisted Crystallization

After alkaline activation and addition of Cu(NO_3_)_2_ and/or TMAdaOH when required, the mixtures were evaporated at 80 °C to form concentrated gels, then dried, ground, and homogenized before crystallization [36]. Vapor-assisted crystallization (VAC/SAC) was performed in a Teflon-lined autoclave, with the dried precursor physically separated from the liquid water and exposed only to generated water vapor [43].

Crystallization was carried out under static conditions at 160 °C for 12 h for the nominal Si/Al = 12 formulations (S1–S4, S10, and S12) and 18 h for the nominal Si/Al = 25 formulations (S5–S8 and S11). After crystallization, the solids were washed with deionized water to approximately pH 9 and dried at 100 °C for 12 h.

### 3.6. Calcination and Conversion to the Protonic Form

The dried crystallization products were calcined in air to 550 °C at 2 °C min^−1^ and held for 6 h. The calcined materials were converted to the ammonium form by three successive ion-exchange cycles in 1.0 mol L^−1^ NH_4_Cl at 80 °C for 2 h per cycle, with washing between cycles. The resulting NH_4_^+^-exchanged materials were then calcined under the same conditions to obtain the protonic form prior to post-synthetic Cu ion exchange.

### 3.7. Post-Synthetic Cu Ion Exchange and Compositional Analysis

All S1–S8 materials were subjected to the same post-synthetic Cu ion-exchange procedure after conversion to the protonic form; thus, the terms Cu-free and Cu-containing crystallization refer only to the crystallization stage. Cu exchange was performed by treating 3.0 g of proton-form zeolite with 100 mL of 0.10 mol L^−1^ Cu(NO_3_)_2_ at 80 °C for 2 h, following Gao et al. [37]. The solids were then washed, dried at 100 °C for 12 h, and calcined at 550 °C for 6 h. The same procedure was applied to S10–S12.

Final bulk Si/Al ratios, Cu contents, and Cu/Al ratios of S1–S8 were determined by ICP-OES. Initial Cu/Al values refer to Cu introduced before crystallization, whereas final Cu/Al values correspond to the post-exchange catalysts and were treated as distinct compositional descriptors.

### 3.8. Integrated Characterization Strategy

The S1–S8 catalyst matrix was characterized by X-ray diffraction (XRD), ^27^Al and ^29^Si magic-angle-spinning nuclear magnetic resonance spectroscopy (MAS NMR), ultraviolet–visible diffuse reflectance spectroscopy (UV–Vis DRS), inductively coupled plasma optical emission spectroscopy (ICP-OES), and ammonia temperature-programmed desorption (NH_3_-TPD). The complete analytical matrix is provided in Table S1.

XRD patterns were recorded using a PANalytical X’Pert Pro diffractometer (PANalytical B.V., Almelo, The Netherlands) with Cu Kα radiation (λ = 1.5406 Å) over 2θ = 5–50°, with a step size of 0.02° and a scan rate of 2° min^−1^. For comparative analysis, the full width at half maximum (FWHM) was determined for the six principal CHA reflections at approximately 2θ = 9.5°, 13.0°, 16.0°, 20.7°, 25.0°, and 30.0°, and the mean FWHM was calculated for each sample. Because no crystallinity standard was applied, the FWHM values were used only as comparative descriptors of reflection breadth and were not converted to crystallite size or phase fraction.

Solid-state ^27^Al and ^29^Si MAS NMR spectra were acquired at 14.1 T using a Bruker BioSpin spectrometer (Bruker BioSpin, Rheinstetten, Germany) spinning rate of 14 kHz. The ^27^Al chemical shifts were referenced to 0.1 mol L^−1^ aqueous Al(NO_3_)_3_ at 0 ppm and the ^29^Si shifts to tetramethylsilane at 0 ppm. For quantitative comparison, the ^27^Al spectra were corrected using a linear baseline and integrated over 40–70, 15–40, and −15–15 ppm, corresponding to the Al(IV), intermediate non-tetrahedral, and Al(VI) regions, respectively. The ^29^Si spectra were fitted using four constrained Gaussian components centered approximately at −98, −103 to −104, −108 to −109, and −114 ppm, with relative contributions calculated from their integrated areas. Because of spectral overlap, these fitted components were treated as comparative spectral contributions rather than assigned uniquely to individual Q^4^(mAl) populations.

UV–Vis diffuse reflectance spectra of the final Cu-containing S1–S8 catalysts were recorded at room temperature over 200–900 nm using a Lambda 650 spectrophotometer (PerkinElmer, Waltham, MA, USA), BaSO_4_ as the white reference and transformed using the Kubelka–Munk function, F(R∞). For quantitative comparison, the transformed spectra were baseline-corrected within the 220–400 and 550–850 nm intervals and integrated numerically; the wavelength of maximum response in the principal UV region was also determined. These descriptors were used comparatively and not as absolute measures of individual Cu species, coordination environments, or oxidation states.

Bulk Si, Al, and Cu contents were determined for S1–S8 using a PerkinElmer Optima 8300 ICP-OES instrument (PerkinElmer, Waltham, MA, USA) after microwave-assisted digestion using a CEM microwave digestion system (CEM Corporation, Matthews, NC, USA) with HNO_3_, HCl, and HF. These acids were obtained from a Merck/Sigma-Aldrich distributor in Porto Alegre, Brazil. Certified calibration standards were used to calculate bulk Si/Al ratios, Cu contents, and molar Cu/Al ratios.

NH_3_-TPD measurements were performed using an AutoChem II system (Micromeritics, Norcross, GA, USA) on 50 mg of the final Cu-exchanged catalysts. Samples were pretreated at 500 °C for 1 h under He, cooled to 160 °C, exposed for 2 h to 500 ppm NH_3_ in He, and purged for 8 h at 160 °C with He containing 2.5 vol% H_2_O. Desorption was then monitored under He from 160 to 800 °C at 10 °C min^−1^ using a total flow rate of 50 mL min^−1^ [34]. For quantitative comparison, the profiles were corrected using a linear baseline between 160 and 800 °C and integrated over 160–410, 410–618.5, and 618.5–800 °C; the interval boundaries were defined from minima in the mean S1–S8 desorption profile. The contribution of each interval was expressed relative to the total integrated signal, and T_max_ values were assigned only when a resolved local maximum was present. Because no absolute NH_3_ calibration was applied, the integrated values are reported as relative signal areas rather than absolute acid-site concentrations.

### 3.9. NH_3_-SCR Catalytic Tests

**Figure 12:**
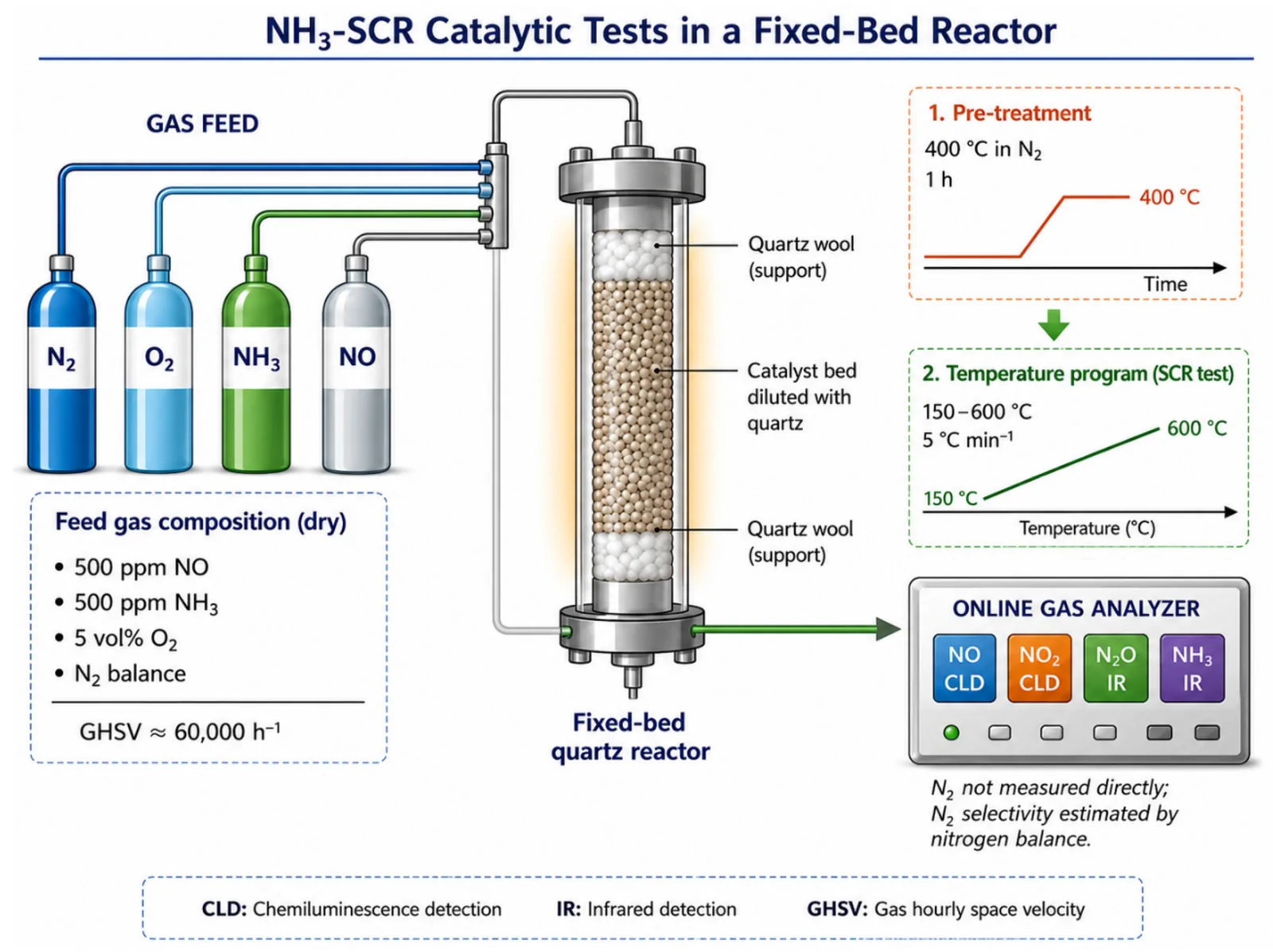
Fixed-bed reactor configuration, dry-feed composition, pretreatment procedure, and temperature program used for comparative NH_3_-SCR light-off tests. N_2_ was not measured directly; estimated N_2_ selectivity was obtained from an atomic nitrogen balance, whereas relative N_2_O formation was expressed separately as an inlet-NO_x_-normalized response.

NH_3_-SCR performance was evaluated by temperature-programmed light-off tests in a continuous-flow fixed-bed quartz reactor (Figure 12). For each test, 100 mg of catalyst with a particle-size fraction of 250–355 μm was physically mixed with 400 mg of inert quartz and loaded into a quartz reactor with an internal diameter of 6 mm. The dry feed consisted of 500 ppm NO, 500 ppm NH_3_, 5 vol% O_2_, and N_2_ as the balance gas at a total flow rate of 100 mL min^−1^, corresponding to a nominal GHSV of approximately 60,000 h^−1^ based on an apparent undiluted catalyst volume of 0.10 cm^3^.

Before each test, the catalyst was pretreated under flowing N_2_ at 400 °C for 1 h. The temperature-programmed NH_3_-SCR response was then recorded from 150 to 600 °C at 5 °C min^−1^. Outlet NO, NO_2_, N_2_O, and NH_3_ concentrations were monitored using an online multicomponent gas analyzer; NO and NO_2_ were measured by chemiluminescence, whereas N_2_O and NH_3_ were measured by infrared detection. The analyzer was calibrated using certified gas mixtures. The resulting profiles were treated as comparative temperature-programmed responses rather than steady-state kinetic measurements.

NO_x_ conversion was determined from the inlet and outlet NO and NO_2_ concentrations. Because N_2_ was not measured directly, estimated N_2_ selectivity was obtained from an atomic nitrogen balance using the measured NO, NO_2_, NH_3_, and N_2_O concentrations, whereas relative N_2_O formation was independently normalized to inlet NO_x_. The complete equations, definitions, and calculation procedures for these nitrogen-product descriptors are provided in Section S1 of the Supplementary Materials.

H-S4 was retained as an additional protonic reference in the fresh NO_x_-conversion comparison but was excluded from the estimated N_2_-selectivity and relative N_2_O-formation analyses because its NO_x_ conversion remained below approximately 1%, making the corresponding nitrogen-balance calculations highly sensitive to small analytical variations.

Apparent T_50_ and T_90_ values were determined by linear interpolation between the experimental temperature points immediately surrounding 50 and 90% NO_x_ conversion on the ascending light-off branch. Hydrothermal-aging effects were further compared using the shift in apparent T_50_ (ΔT_50_) and the retained maximum NO_x_ conversion, defined as the ratio of the maximum aged to fresh NO_x_ conversion, expressed as a percentage. Maximum conversion corresponded to the highest measured value within the 150–600 °C test range. These parameters were used exclusively as comparative descriptors of catalytic response and not as kinetic parameters.

### 3.10. Hydrothermal Aging

Accelerated hydrothermal aging was performed on the final Cu-containing catalyst matrix in a tubular quartz reactor with an internal diameter of 8 mm using 200 mg of catalyst (250–355 μm). The catalysts were exposed to synthetic air containing 10 vol% H_2_O at a total flow rate of 200 mL min^−1^. Water was delivered by a syringe pump and vaporized before entering the reactor. The temperature was increased from room temperature to 750 °C at 5 °C min^−1^ and maintained at 750 °C for 16 h under the humidified synthetic-air flow, followed by natural cooling to room temperature under the same atmosphere.

The aged catalysts were subsequently evaluated by temperature-programmed NH_3_-SCR testing under the same conditions applied to the corresponding fresh materials. Fresh-versus-aged comparisons were based on the catalytic descriptors defined in Section 3.9. The aging treatment was used as an accelerated comparative hydrothermal exposure, and the resulting changes were interpreted in terms of retained catalytic functionality under the applied conditions.

## 4. Conclusions

This study demonstrates that rice husk ash and metakaolin can be used as biomass-waste-derived and mineral-derived precursors for the preparation of Cu-SSZ-13 catalysts through solvent-lean vapor-assisted crystallization under OSDA-free and reduced-OSDA conditions. Across the S1–S8 matrix, XRD showed predominant CHA formation, ^27^Al MAS NMR indicated predominantly tetrahedral framework Al, and ^29^Si MAS NMR revealed systematic differences in the average silicate-framework environment.

After post-synthetic Cu exchange, final Cu contents ranged from 1.50 to 2.61 wt% and Cu/Al ratios from 0.22 to 0.27. UV–Vis DRS and NH_3_-TPD revealed corresponding differences in Cu-related optical response and NH_3_-interaction behavior, although neither descriptor alone accounted for the catalytic trends across the complete matrix. Fresh NH_3_-SCR apparent T_50_ values ranged from 201.3 to 278.4 °C, with maximum NO_x_ conversions of 89.9–97.0%; S4 exhibited the earliest light-off and the highest maximum conversion.

Accelerated hydrothermal aging shifted apparent T_50_ by 32.4–65.3 °C while retaining 91.1–94.9% of the fresh maximum NO_x_-conversion response. Estimated N_2_ selectivity remained high throughout most of the principal activity region, whereas relative N_2_O formation remained limited and increased mainly at elevated temperature. These results indicate that the principal effect of aging was delayed catalytic activation rather than a major loss of maximum conversion or a large alteration of the overall nitrogen-product response.

Overall, the results support a comparative structure–property–function relationship in which framework composition, final Cu incorporation, NH_3_ interaction, synthesis history, and transport collectively determine NH_3_-SCR behavior. The main contribution of the work is therefore not catalytic superiority over optimized Cu-SSZ-13 systems, but the demonstration that an RHA/MK-derived, OSDA-free or reduced-OSDA, solvent-lean synthesis platform can produce functional Cu-SSZ-13 materials with substantial dry-feed NH_3_-SCR activity and retained functionality after accelerated hydrothermal exposure.

Future work should address wet-feed NH_3_-SCR operation, replicated steady-state testing, longer-term hydrothermal exposure, post-aging structural characterization, and more detailed investigation of the relationships among synthesis-stage Cu chemistry, framework organization, and final Cu environments.

## Figures and Tables

**Table 1:** Quantitative integration of the baseline-corrected ^27^Al MAS NMR spectra of S1–S8 using common chemical-shift regions.

Sample	Al(IV) Region (%)	Intermediate Region (%)	Al(VI) Region (%)	δmax Al(IV) (ppm)	δmax Intermediate (ppm)	δmax Al(VI) (ppm)
S1	83.06	8.38	8.56	55.0	28.0	−0.1
S2	84.68	7.65	7.66	55.1	28.1	0.3
S3	82.40	8.85	8.75	54.8	28.2	0.1
S4	84.06	7.93	8.01	54.9	28.3	0.2
S5	82.33	8.86	8.81	55.0	28.5	0.1
S6	84.69	7.77	7.54	55.0	28.1	−0.1
S7	83.29	8.38	8.33	54.8	28.3	−0.3
S8	85.47	7.35	7.18	54.9	28.3	−0.3

Notes: The ^27^Al MAS NMR spectra were baseline-corrected using a linear baseline across the analyzed spectral range. Common integration intervals of 40–70 ppm, 15–40 ppm, and −15–15 ppm were applied to all samples and are reported as the Al(IV), intermediate non-tetrahedral, and Al(VI) regions, respectively. Regional contributions are expressed as percentages of the summed integrated signal within these three intervals. The intermediate region is treated as a composite non-tetrahedral contribution and is not assigned uniquely to a single Al coordination state.

**Table 2:** Relative integrated contributions of the four fitted components obtained from the ^29^Si MAS NMR spectra of S1–S8.

Sample	Component Near −98 ppm (%)	Component Near −103 to −104 ppm (%)	Component Near −108 to −109 ppm (%)	Component Near −114 ppm (%)
S1	7.9	18.8	45.8	27.5
S2	5.9	20.3	46.4	27.4
S3	6.7	20.8	46.1	26.4
S4	5.1	20.4	47.9	26.6
S5	7.3	11.2	24.4	57.1
S6	5.0	10.9	29.9	54.2
S7	5.4	11.7	27.2	55.7
S8	3.4	11.3	32.0	53.3

Notes: Relative contributions were calculated from the integrated areas of four constrained Gaussian components fitted to the ^29^Si MAS NMR spectral envelopes. The component positions are reported as spectral regions and are used for comparative purposes only. Because of spectral overlap and possible contributions from defect-related Si environments, particularly in the −103 to −104 ppm region, the fitted components were not assigned uniquely to individual Q^4^(mAl) species.

**Table 3:** Quantitative comparative analysis of the baseline-corrected NH_3_-TPD profiles of the final Cu-exchanged S1–S8 catalysts.

Sample	Total Integrated Area (a.u.·°C)	Low-Temperature Contribution (%)	Intermediate-Temperature Contribution (%)	High-Temperature Contribution (%)	T_max_, Low-Temperature Region (°C)	T_max_, Intermediate-Temperature Region (°C)	T_max_, High-Temperature Region (°C)
S1	27.05	37.6	38.3	24.1	309.5	527.0	674.0
S2	27.70	37.9	37.4	24.7	311.5	517.5	682.5
S3	27.83	31.5	33.9	34.7	312.0	521.5	689.5
S4	29.81	28.6	33.1	38.3	310.5	526.0	684.0
S5	30.26	38.6	42.9	18.5	309.0	522.0	—
S6	31.34	38.9	42.5	18.6	309.5	519.5	—
S7	34.21	38.3	44.2	17.5	310.5	515.5	—
S8	35.23	39.5	44.6	16.0	311.0	518.0	—

Notes: NH_3_-TPD profiles were baseline-corrected using a linear baseline between 160 and 800 °C and integrated numerically. The common integration ranges were 160–410 °C, 410–618.5 °C, and 618.5–800 °C for the low-, intermediate-, and high-temperature regions, respectively. Percentage contributions correspond to the fraction of the total integrated signal within each temperature interval. Because the NH_3_-TPD signal was not calibrated for absolute NH_3_ uptake, the integrated values are reported as relative integrated areas and should not be interpreted as absolute acid-site concentrations. For S5–S8, no clearly resolved local maximum was identified within the high-temperature region; therefore, T_max_ was not assigned.

**Table 4:** Quantitative NH_3_-SCR light-off and maximum-conversion descriptors of the S1–S8 catalysts before and after accelerated hydrothermal aging.

Sample	T_50_ Fresh (°C)	T_50_ Aged (°C)	ΔT_50_ (°C)	Maximum NO_x_ Conversion Fresh (%)	Maximum NO_x_ Conversion Aged (%)	Retained Maximum NO_x_ Conversion (%)
S1	237.6	269.9	32.4	93.3	88.6	94.9
S2	221.6	263.5	41.9	95.3	89.1	93.5
S3	268.3	302.4	34.1	91.8	83.7	91.1
S4	201.3	266.6	65.3	97.0	89.2	91.9
S5	248.0	283.0	35.0	92.4	87.4	94.6
S6	241.0	278.4	37.4	93.1	87.5	94.0
S7	278.4	312.8	34.4	89.9	82.1	91.3
S8	238.4	280.6	42.2	93.6	87.7	93.6

Notes: T_50_ values were determined by linear interpolation between the experimental temperature points surrounding 50% NO_x_ conversion on the ascending light-off branch. ΔT_50_ was calculated as T_50_,aged − T_50_,fresh. Maximum NO_x_ conversion corresponds to the highest measured conversion within the 150–600 °C temperature-programmed test range. Retained maximum NO_x_ conversion was calculated as the ratio of the aged to fresh maximum conversion, expressed as a percentage.

**Table 5:** Final bulk Si/Al ratios, Cu contents, and molar Cu/Al ratios of the post-synthetically Cu-exchanged S1–S8 catalysts.

Sample	Final Si/Al Ratio	Final Cu Content (wt%)	Final Molar Cu/Al Ratio	Synthesis Condition/Sample Role
S1	12.3	2.32	0.22	Cu-free crystallization, OSDA-free
S2	12.4	2.39	0.23	Cu-containing crystallization, OSDA-free
S3	12.2	2.50	0.25	Cu-free crystallization, OSDA-lean
S4	12.1	2.61	0.27	Cu-containing crystallization, OSDA-lean
S5	24.8	1.50	0.23	Cu-free crystallization, OSDA-free
S6	24.7	1.55	0.24	Cu-containing crystallization, OSDA-free
S7	25.0	1.64	0.22	Cu-free crystallization, OSDA-lean
S8	25.2	1.72	0.23	Cu-containing crystallization, OSDA-lean

Note: “Cu-free crystallization” and “Cu-containing crystallization” refer exclusively to the absence or presence of Cu during the crystallization stage. All S1–S8 samples were subsequently subjected to the same post-synthetic Cu ion-exchange procedure.

**Table 6:** Literature comparison of NH_3_-SCR performance and preparation strategies of representative Cu-SSZ-13 systems and the present RHA/MK-derived catalysts.

Catalyst/Study	Preparation Strategy and Synthesis Complexity	Representative NH_3_-SCR Performance	Comparison with the Present Work
S1–S8, present work	RHA/MK-derived route; solvent-lean VAC; OSDA-free or OSDA-lean formulations; common post-synthetic Cu ion exchange	Fresh T_50_ = 201.3–278.4 °C; maximum NO_x_ conversion = 89.9–97.0%	Combines biomass-waste-derived silica, reduced or zero OSDA demand, and solvent-lean crystallization with substantial NH_3_-SCR functionality
Xie et al. [12]	One-pot Cu-SSZ-13 synthesis with direct Cu incorporation during synthesis	High NH_3_-SCR activity and N_2_ selectivity over approximately 150–550 °C	Earlier activation and broader high-performance temperature window than the present catalyst matrix, using an optimized one-pot synthesis route
Lv et al. [18]	Al-rich Cu-SSZ-13 prepared through different synthesis strategies; synthesis conditions tailored to control Al distribution and hydrothermal stability	High NH_3_-SCR activity together with strong hydrothermal stability; catalytic behavior strongly depended on synthesis route	Demonstrates that optimized control of synthesis and Al distribution can provide stronger catalytic and hydrothermal performance, although with a more specifically engineered preparation strategy
Robles et al. [19]	Post-synthetically treated SSZ-13 followed by Cu modification; additional post-synthesis processing steps	Effective NH_3_-SCR-DeNO_x_ activity after structural modification and Cu incorporation	Shows that post-synthetic optimization can enhance catalytic functionality, but requires additional treatment steps after zeolite synthesis
Yang et al. [29]	Hard-template-assisted trans-crystallization synthesis of hierarchical Cu-SSZ-13; additional templating and trans-crystallization steps	Enhanced low-temperature NH_3_-SCR activity and improved hydrothermal stability	Superior low-temperature behavior achieved through hierarchical pore engineering, but with a more complex preparation sequence than the present VAC route
Wang et al. [35]	One-pot synthesis of Na^+^-free Cu-SSZ-13; direct preparation without a separate Na^+^-removal step	Strong NH_3_-SCR performance combined with hydrothermal stability	Representative efficient one-pot route with high catalytic performance and reduced post-synthesis ion-exchange requirements
Al Jabri et al. [36]	Dry-gel conversion synthesis of Cu/SSZ-13; low-liquid crystallization followed by Cu incorporation	High NH_3_-SCR performance and hydrothermal stability for the DGC-derived catalyst	Particularly relevant benchmark because it combines a low-liquid preparation strategy with high catalytic performance
Gao et al. [37]	Conventional SSZ-13 synthesis followed by controlled Cu ion exchange at different Cu loadings	High standard and fast NH_3_-SCR activity associated with Cu loading, location, and accessibility	Provides a fundamental high-performance structure–activity benchmark, although based on conventional zeolite preparation followed by controlled Cu exchange

## Data Availability

The raw and processed data supporting the findings of this study are retained by the authors and are available from the corresponding author upon reasonable request, subject to applicable institutional data-sharing procedures. The retained raw datasets include XRD, ^27^Al and ^29^Si MAS NMR, UV–Vis DRS, ICP-OES, NH_3_-TPD, and NH_3_-SCR catalytic-testing data.

## Supplementary Materials

The following supporting information can be downloaded at: https://www.mdpi.com/article/10.3390/molecules31183282/s1, Table S1: Experimental synthesis matrix, sample roles, characterization, and functional evaluation of the SSZ-13 materials; Table S2: Detailed precursor quantities, nominal synthesis compositions, and crystallization conditions used for the S1–S8 and auxiliary S10–S12 preparations; Section S1: Calculation of NH_3_-SCR Nitrogen-Product Descriptors.
