# Supplementary material for: Biomass-Waste-Derived Silica for Solvent-Lean Cu-SSZ-13 Porous Catalysts in NH3-SCR NOx Remediation
Source: Molecules. 2026 Sep 16;31(18):3282. doi: 10.3390/molecules31183282 (PMC13610021; doi:10.3390/molecules31183282)
Supplement: Supplementary file 1 [file molecules-31-03282-s001.zip › molecules-4527754-supplementary.pdf]

Supplementary Materials

Biomass-Waste-Derived Silica for Solvent-Lean Cu-SSZ-13 Porous Catalysts in NH_3-SCR NO_x Remediation

Table S1. Experimental synthesis matrix, sample roles, characterization, and functional evaluation of the SSZ-13 materials.

Sample	Precursor system	Nominal Si/Al	OSDA condition	Cu during crystallization	Experimental role	Main characterization	Fresh NH_3-SCR	HTA	Aged NH_3-SCR
**S1**	RHA/MK	12	OSDA-free	No	Cu-free member of S1/S2	XRD; ^{27}Al and ^{29}Si MAS NMR; UV–Vis DRS; ICP-OES; NH_3-TPD	Yes	Yes	Yes
**S2**	RHA/MK	12	OSDA-free	Yes	Cu-containing member of S1/S2	XRD; ^{27}Al and ^{29}Si MAS NMR; UV–Vis DRS; ICP-OES; NH_3-TPD	Yes	Yes	Yes
**S3**	RHA/MK	12	OSDA-lean	No	Cu-free member of S3/S4	XRD; ^{27}Al and ^{29}Si MAS NMR; UV–Vis DRS; ICP-OES; NH_3-TPD	Yes	Yes	Yes
**S4**	RHA/MK	12	OSDA-lean	Yes	Cu-containing member of S3/S4	XRD; ^{27}Al and ^{29}Si MAS NMR; UV–Vis DRS; ICP-OES; NH_3-TPD	Yes	Yes	Yes
**S5**	RHA/MK	25	OSDA-free	No	Cu-free member of S5/S6	XRD; ^{27}Al and ^{29}Si MAS NMR; UV–Vis DRS; ICP-OES; NH_3-TPD	Yes	Yes	Yes
**S6**	RHA/MK	25	OSDA-free	Yes	Cu-containing member of S5/S6	XRD; ^{27}Al and ^{29}Si MAS NMR; UV–Vis DRS; ICP-OES; NH_3-TPD	Yes	Yes	Yes
**S7**	RHA/MK	25	OSDA-lean	No	Cu-free member of S7/S8	XRD; ^{27}Al and ^{29}Si MAS NMR; UV–Vis DRS; ICP-OES; NH_3-TPD	Yes	Yes	Yes
**S8**	RHA/MK	25	OSDA-lean	Yes	Cu-containing member of S7/S8	XRD; ^{27}Al and ^{29}Si MAS NMR; UV–Vis DRS; ICP-OES; NH_3-TPD	Yes	Yes	Yes
**S10**	RHA/MK	12	OSDA-lean	Yes	Additional preparation corresponding to S4	Not included in detailed structural or functional matrix	No	No	No
**S11**	RHA/MK	25	OSDA-lean	Yes	Additional preparation corresponding to S8	Not included in detailed structural or functional matrix	No	No	No
**S12**	RHA/MK	12	OSDA-free	No	$NaNO_3$-containing salt control corresponding to S2	Not included in detailed structural or functional matrix	No	No	No

Notes: RHA, rice husk ash; MK, metakaolin; OSDA, organic structure-directing agent; XRD, X-ray diffraction; MAS NMR, magic-angle-spinning nuclear magnetic resonance spectroscopy; UV–Vis DRS, ultraviolet–visible diffuse reflectance spectroscopy; ICP-OES, inductively coupled plasma optical emission spectroscopy; NH_3-TPD, ammonia temperature-programmed desorption; NH_3-SCR, selective catalytic reduction of NO_x with ammonia; HTA, accelerated hydrothermal aging. 'Cu-free' and 'Cu-containing' refer exclusively to the crystallization stage; all S1–S8 catalysts underwent the same post-synthetic Cu ion-exchange procedure before catalytic evaluation.

Table S2. Detailed precursor quantities, nominal synthesis compositions, and crystallization conditions used for the S1–S8 and auxiliary S10–S12 preparations.

Sample	Precursor system	RHA (g)	MK (g)	NaOH (mol L^{-1})	Initial alkaline solution (mL)	TMAdaOH/Si	Nominal atomic Si/Al	Initial Cu/Al	$Cu(NO_3)_2$ solution (mol L^{-1})	$Cu(NO_3)_2$ solution volume (mL)	$NaNO_3$-containing salt-control additive	VAC time (h)
S1	RHA/MK	8.40	1.60	1.0	70	0	12	0	—	—	—	12
S2	RHA/MK	8.40	1.60	1.0	70	0	12	0.047	0.07	8.5	—	12
S3	RHA/MK	8.40	1.60	1.0	70	0.035	12	0	—	—	—	12
S4	RHA/MK	8.40	1.60	1.0	70	0.035	12	0.047	0.07	8.5	—	12
S5	RHA/MK	11.05	0.95	1.0	70	0	25	0	—	—	—	18
S6	RHA/MK	11.05	0.95	1.0	70	0	25	0.041	0.07	4.5	—	18
S7	RHA/MK	11.05	0.95	1.0	70	0.035	25	0	—	—	—	18
S8	RHA/MK	11.05	0.95	1.0	70	0.035	25	0.041	0.07	4.5	—	18
S10	RHA/MK	8.40	1.60	1.0	70	0.035	12	0.047	0.07	8.5	—	12
S11	RHA/MK	11.05	0.95	1.0	70	0.035	25	0.041	0.07	4.5	—	18
S12	RHA/MK	8.40	1.60	1.0	70	0	12	0	—	—	$NaNO_3$, 1.79 mmol	12

Notes: RHA, rice husk ash; MK, metakaolin; TMAdaOH, tetramethyladamantanammonium hydroxide; VAC, vapor-assisted crystallization. Initial Cu/Al, $Cu(NO_3)_2$ concentration, and $Cu(NO_3)_2$ solution volume refer to Cu introduced during the crystallization stage and do not represent the final Cu content after post-synthetic ion exchange. S12 contains 1.79 mmol $NaNO_3$ as the salt-control formulation corresponding to S2.

Section S1. Calculation of NH_3-SCR Nitrogen-Product Descriptors

NO_x conversion was calculated from the inlet and outlet concentrations of NO and NO_2 according to:

$$X_{\mathrm{NO_x}}(\%) = \frac{\left(C_{\mathrm{NO,in}} + C_{\mathrm{NO_2,in}}\right) - \left(C_{\mathrm{NO,out}} + C_{\mathrm{NO_2,out}}\right)}{C_{\mathrm{NO,in}} + C_{\mathrm{NO_2,in}}} \times 100$$

where $C_{\mathrm{NO,in}}$ and $C_{\mathrm{NO_2,in}}$ are the inlet concentrations of NO and NO_2, respectively, and $C_{\mathrm{NO,out}}$ and $C_{\mathrm{NO_2,out}}$ are the corresponding outlet concentrations. Because NO_2 was not introduced in the inlet feed, $C_{\mathrm{NO_2,in}}$ was taken as zero.

Because N_2 was not measured directly, its contribution was estimated from an atomic nitrogen balance using the measured inlet and outlet concentrations of NO, NO_2, NH_3, and N_2O. The reacted nitrogen was calculated as:

$$N_{\mathrm{reacted}} = \left(C_{\mathrm{NO,in}} + C_{\mathrm{NO_2,in}} + C_{\mathrm{NH_3,in}}\right) - \left(C_{\mathrm{NO,out}} + C_{\mathrm{NO_2,out}} + C_{\mathrm{NH_3,out}}\right)$$

where $C_{\mathrm{NH_3,in}}$ and $C_{\mathrm{NH_3,out}}$ are the inlet and outlet NH_3 concentrations, respectively.

Estimated N_2 selectivity was calculated by assigning the reacted nitrogen not detected as N_2O to molecular nitrogen:

$$S_{\mathrm{N_2,est}}(\%) = \frac{N_{\mathrm{reacted}} - 2\left(C_{\mathrm{N_2O,out}} - C_{\mathrm{N_2O,in}}\right)}{N_{\mathrm{reacted}}} \times 100$$

The resulting values are reported as estimated N_2 selectivity because N_2 was not measured directly. Any unmeasured nitrogen-containing products, if present, would contribute to the residual nitrogen balance and could therefore affect the estimated N_2 contribution.

The temperature-dependent N_2O response was expressed independently as relative N_2O formation:

$$F_{\mathrm{N_2O,rel}}(\%) = \frac{2(C_{\mathrm{N_2O,out}} - C_{\mathrm{N_2O,in}})}{C_{\mathrm{NO,in}} + C_{\mathrm{NO_2,in}}} \times 100$$

where the factor of two accounts for the two nitrogen atoms in each N_2O molecule. Because N_2O was not introduced in the inlet feed, $C_{\mathrm{N_2O,in}}$was taken as zero. Relative N_2O formation is normalized to inlet NO_x and is therefore not equivalent to N_2O selectivity based on reacted nitrogen. Estimated N_2 selectivity and relative N_2O formation have different denominators and were not treated as complementary fractions of a closed nitrogen balance or constrained to sum to 100%.

These nitrogen-product descriptors were evaluated for the S1–S8 catalysts in both fresh and hydrothermally aged states only within temperature regions where the relevant denominators were sufficiently above analytical uncertainty. H-S4 was excluded from the estimated N_2-selectivity and relative N_2O-formation analyses because its NO_x conversion remained below approximately 1%, making the corresponding nitrogen-balance calculations numerically unstable and sensitive to small analytical variations.
